# Cohesin acetylation and ATPase activity control cohesion and loop architecture through distinct mechanisms

**DOI:** 10.1073/pnas.2531218123

**Published:** 2026-04-22

**Authors:** Lorenzo Costantino, Tiantian Ye, Kevin Boardman, Siheng Xiang, Jonathan Luo, Yudi Mu, Wenxiu Ma, Douglas Koshland

**Affiliations:** ^a^Department of Molecular and Cell Biology, University of California Berkeley, Berkeley, CA 94720; ^b^Research Institute of Molecular Pathology, Vienna BioCenter, Vienna 1030, Austria; ^c^Department of Statistics, University of California Riverside, Riverside, CA 92521

**Keywords:** cohesin, chromatin loops, cohesion, genome structure, ATPase

## Abstract

Cohesin is a conserved protein complex that mediates sister chromatid cohesion, chromosome condensation, gene regulation, and DNA repair. These processes rely on cohesin’s ability to tether DNA and form chromatin loops, which depend on cohesin’s ATP hydrolysis activity and Eco1-mediated acetylation of two lysines (K112 and K113 in budding yeast) in its Smc3 subunit. However, how cohesin’s ATPase activity and acetylation integrate to control cohesin functions in vivo remains poorly understood. To address this, we analyzed chromatin architecture in yeast mutants with altered cohesin acetylation, and/or ATPase activity. Single K112 or K113 acetyl-null mutants retained wild-type loop length distributions and positioned loops at cohesin-associated regions (CARs), suggesting acetylation of either lysine alone is sufficient for loop positioning. Conversely, Eco1 depletion (removing both acetylations) led to extended loops and loss of positioned loops, despite unchanged cohesin binding. We found that a cohesin acetylation mutant lacking the tethering activity required for cohesion could form positioned loops like the wild type, whereas cohesion-competent mutants lacked positioned loops. Together, these results support a model in which cohesin’s activities required for cohesion and loop formation are mechanistically separable, arguing against a passive loop-capture mechanism. K112 acetyl-mimic mutant partially reduced ATPase activity, yet showed wild-type loop profile, suggesting that lowering ATPase activity does not dictate loop positioning. However, hyper-ATPase mutants exhibited fewer random loops and more positioned loops, indicating that elevated ATPase promotes loop stabilization. Together, these results indicate that acetylation fine-tunes cohesin ATPase activity and function to shape genome architecture.

## Significance

The DNA within chromosomes is organized into higher-order structures that support proper chromosome inheritance and gene expression. The conserved protein complex cohesin plays a central role in establishing these structures in all eukaryotes by extruding DNA loops and tethering different chromosomal regions, both within and between chromosomes. Defects in cohesin function are associated with cancer and birth defects. In this study, we investigate how cohesin’s loop extrusion and tethering activities are regulated using a panel of budding yeast mutants that affect cohesin or its associated factors. Our findings provide insights into the distinct regulation of cohesin looping and tethering activities by cohesin’s ATPase activity and its acetylation.

**Fig. 1.**
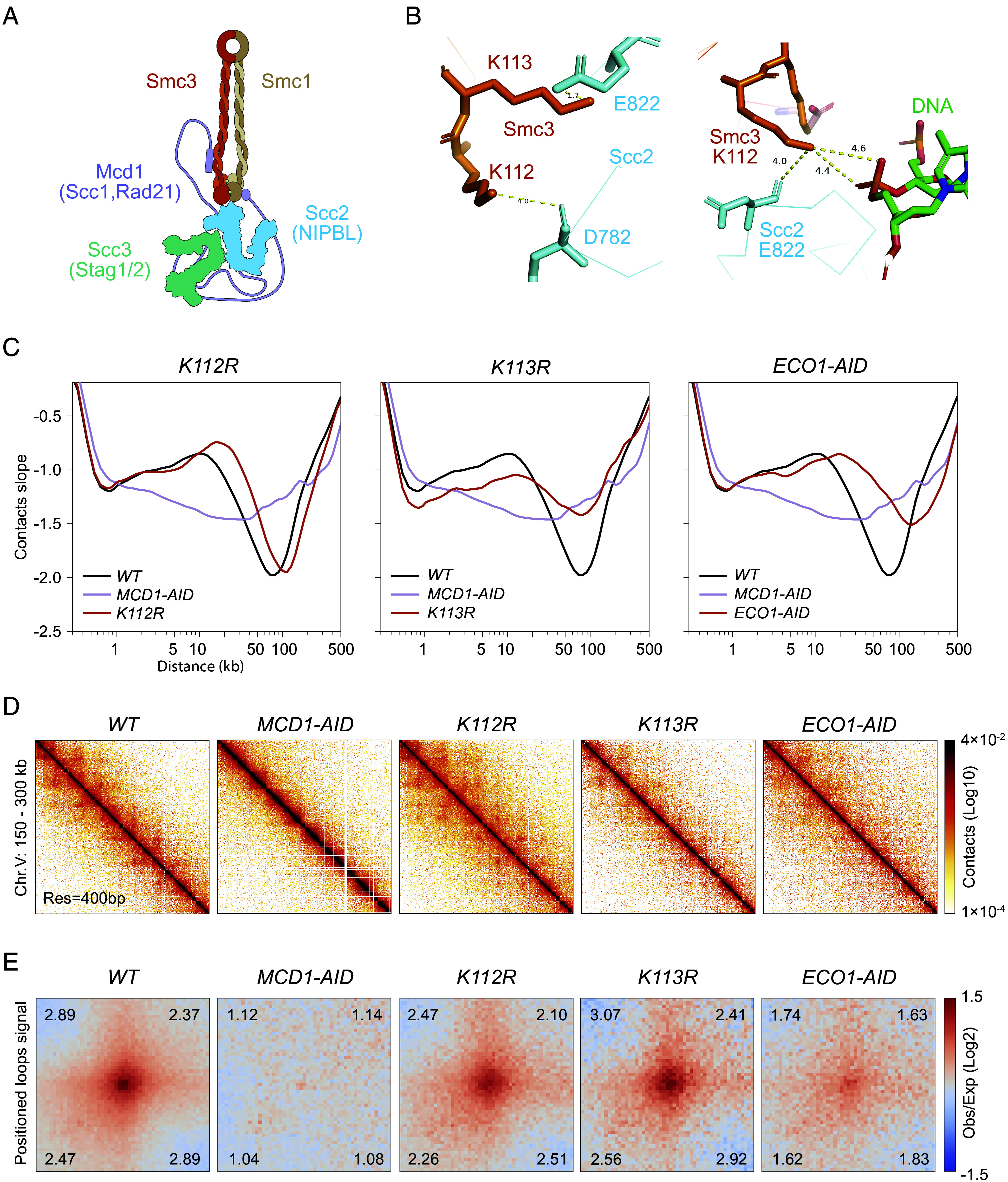
Chromosome structure in wild-type and cohesin acetylation mutants. (*A*) Cartoon depiction of the cohesin complex bound by the loader subunit Scc2p. Protein subunit names shown are from budding yeast, with alternative names for yeast (Scc1) and human orthologs (Rad21, NIPBL, Stag1/2) indicated in parentheses. (*B*) Cryo-EM structure of *S. cerevisiae* cohesin (PDB ID: 6ZZ6). The *Left* panel highlights Smc3-K113 proximity to Scc2-E822; the *Right* panel shows Smc3-K112 proximity to DNA and Scc2-D782. (*C*) Chromosome contacts in cohesin acetylation mutants. Micro-C XL analysis of chromosome interactions in mitotically arrested wild-type cells (*WT*), single acetylation-deficient mutants smc3-K112R (*K112R*) and smc3-K113R (*K113R*), and cells depleted of Eco1 acetyltransferase (*ECO1-AID*) or cohesin (*MCD1-AID*). Derivative slopes of the contact frequency curves (shown in *SI Appendix*, Fig. S1*A*) are plotted against genomic distance in black for *WT,* mauve for *MCD1-AID*, and red for acetylation-mutants. The same wild-type and MCD1-AID datasets are shown in all subsequent Micro-C XL figures to facilitate comparison of chromosome architecture in each mutant with either wild-type cells (fully functional cohesin) or MCD1-AID cells (lacking cohesin function). (*D*) Contact maps in cohesin acetylation mutants. Micro-C XL contact maps at 400 bp resolution over an arm region at chromosome V 150 to 300 kb for the indicated strains listed in *C*. Interaction intensity in contact maps will be represented throughout the paper using a standard log10 scale colormap, ranging from white (no detectable interactions) to black (strongest interactions). (*E*) Genome-wide signal for positioned loops at CARs in cohesin acetylation mutants. Piled-up heatmap of the ±5 kb regions centered at CARs for the indicated strains listed in *C*. Numbers in the corners represent the fold-change of the signal enrichment of the center pixel over the indicated corner pixels. The pile-ups throughout the paper will utilize a diverging colormap for observed/expected signal in log2, with positive signal enrichment represented in red and negative signals in blue.

Cohesin is a conserved eukaryotic protein complex (Fig. 1*A*) that mediates sister chromatid cohesion, which is essential for chromosome segregation and cell viability. It also facilitates DNA repair, chromosome condensation, and regulates gene expression. Cohesin defects have been linked to birth defects and various diseases, including cancers (1). For many years, cohesin’s biological activities were believed to stem from its ability to tether two DNA regions within a chromosome or between sister chromatids (2, 3). However, recent in vitro studies have shown that cohesin and other members of the SMC (Structural Maintenance of Chromosomes) family of proteins can processively extrude DNA loops, a process named loop extrusion (4–6). This biochemical activity explains cohesin’s in vivo role in forming and maintaining dynamic, genome-wide intrachromosomal loops detected by chromosome conformation capture techniques (7–11). Tethering and loop extrusion require cohesin’s ATPase activity, and are regulated by Eco1 acetylation of cohesin’s Smc3 subunit (5, 6, 12–15). A deeper understanding of the roles and interplay of cohesin ATPase and acetylation in tethering and looping is crucial for comprehending cohesin’s capacity to promote diverse biological functions.

Cohesin ATPase and acetylation have been intensively studied, revealing a complex relationship. Cohesin forms two ABC-like ATPase active sites through dimerization of its Smc1 and Smc3 subunits (16). The ATP binding and ATPase activity of both active sites are required for cohesin binding to DNA, and hence for all cohesin’s functions, including cell viability (12, 13). Eco1 acetylation of cohesin is also essential for cell viability (14, 15, 17–19). It promotes stable DNA binding (20), the formation of cohesion by tethering the second sister chromatid (21), and the control of loop size (22–24). Eco1p acetylates a conserved lysine in cohesin’s Smc3 subunit (K113 in budding yeast and K106 in mammals) (Fig. 1*B*) (14, 15, 19). Several studies have exploited K113 mutations that block (K113R) or mimic (K113Q) acetylation, suggesting that regulated K113 acetylation is essential for viability and cohesion (14, 15, 19). Follow-up studies indicated that K113 acetylation downregulates cohesin ATPase activity by perturbing its binding to, and therefore activation by Scc2 [Fig. 1*B* and (21, 25, 26)]. Scc2 is necessary for promoting both chromosome binding in vivo and loop extrusion in vitro (5, 6, 27).

Several important questions regarding cohesin’s ATPase and acetylation remain unanswered. For example, establishing the role of cohesin ATPase in DNA binding was critical. However, DNA binding is only the first step in generating cohesion or DNA loops. The contribution of cohesin ATP hydrolysis to subsequent steps, like capturing a second DNA strand or modulating loop size, is unknown. As a second example, Eco1p also acetylates a conserved lysine Smc3-K112 (K105 in mammals) that is adjacent to K113 (Fig. 1*B*) (14, 15, 19). The analysis of K112 acetyl null (K112R) showed that K112 acetylation was not required for viability or cohesion. In the absence of a biological function, the roles of K112 acetylation in DNA binding, ATPase activity, or loop formation have not been investigated. The proximity of K112 to an aspartate on Scc2 and to a phosphate on the DNA backbone (Fig. 1*B*) suggests K112 might also promote Scc2 stimulation of cohesin ATPase in response to DNA binding. If so, does K112 acetylation impact loop size or position? To answer these and related questions, we study the impact of a panel of hypomorphic mutations that alter cohesin ATPase activity or its acetylation on looping and tethering.

## Results

### K112 or K113 Acetylation Regulates Chromatin Loop Size and Positioning.

Eco1p acetylation of cohesin is required to constrain loop size and form positioned loops, since depleting Eco1p or mutating both K112 and K113 to acetyl nulls allows loop expansion (22–24, 28). However, the individual contributions of K112 and K113 acetylation in looping or controlling ATPase levels have not been studied. To understand the role of K112 and K113 acetylation in chromosome structure, we utilized two acetyl-null mutants at these positions (all strain genotypes are listed in *SI Appendix*, Table S1). These strains expressed only a Smc3 subunit that was either blocked for K112 acetylation (smc3-K112R) or K113 acetylation (smc3-K113R SMC3-AID in the presence of auxin). We compared the chromosome architecture in these strains by arresting them in mitosis and subsequently performing Micro-C XL (11, 29). As controls, we also used Micro-C XL to analyze chromosome architecture in Eco1-depleted (ECO1-AID in the presence of auxin) or cohesin-depleted (MCD1-AID in the presence of auxin) cells (*SI Appendix*, Fig. S1*A*). For brevity and clarity, we referred to these strains in the remainder of the text and figure legends as K112R, K113R, ECO1-AID, and MCD1-AID, respectively. We also compared their structures to those found by Hi-C analysis of the smc3-K112R, K113R double mutant, and its corresponding wild type (22).

We began our Micro-C XL analysis by plotting contact decay curves, which show the average frequency of chromatin contacts as a function of genomic distance—that is, how often two genomic regions interact based on their separation (*SI Appendix*, Fig. S1*B*). To more precisely compare strains, we also examined the derivative (slope) of these curves, which captures the rate of change in contact frequency across genomic distances (Fig. 1*C*). As previously shown (11), wild-type cells exhibited both elevated contact frequencies and more positive derivative slopes compared to cohesin-depleted cells, indicating that interactions around the 10 kb range are largely dependent on cohesin (*WT* vs *MCD1-AID* in Fig. 1*C* and *SI Appendix*, Fig. S1*B*). We also observed that yeast cells lacking cohesin K112 and K113 acetylation (*ECO1-AID, K112R-K113R*) exhibited a marked increase in both derivative slopes and contact frequencies in the 50 to 200 kb range compared to wild type, *K112R*, or *K113R* cells [Fig. 1*C*, *SI Appendix*, Fig. S1*B*, and (22, 23)]. These results confirmed that Eco1-mediated acetylation limits the size of chromatin loops and is required for positioned loop formation (22, 30).

Interestingly, the *K112R* mutant closely resembled the wild-type positive slope with enriched contacts in the 20 to 50 kb range, suggesting robust looping activity with slightly longer loops being formed. The *K113R* mutant also displayed a clear gain in contact frequencies and positive slopes relative to cohesin-depleted cells, though somewhat attenuated compared to wild type. These profiles indicate that *K113R* retains substantial loop-forming capacity, suggesting that looping could occur independently of cohesin’s tethering activity, since the *K113R* mutant was defective in sister chromatid cohesion (around 60% defect), as previously reported (14, 15).

In wild-type cells, cohesin-dependent interactions detected by chromosome conformation capture techniques are thought to arise from the crosslinking of sequences, which can occur either within or between sister chromatids (31, 32). In contact maps, the diffuse off-diagonal “fuzz” reflects random looping events with variable anchor positions, whereas distinct off-diagonal “spots” represent positioned loops anchored at specific, recurrent genomic sites that correspond to cohesin-associated regions (CARs) in budding yeast (Fig. 1*D* and *SI Appendix*, Fig. S1*C*). To quantify genome-wide the presence of positioned loops anchored at CARs, we performed a pile-up analysis of contact maps centered on CARs using 5-kilobase flanking regions, which revealed a genome-wide enrichment of signal above the background level of random distal interactions in wild-type (Fig. 1*E*). Strikingly, these off-diagonal spots were also present in the contact maps and pile-ups of the K112R and K113R single mutants, with signal intensities comparable to those observed in wild-type cells (Fig. 1 *D* and *E* and *SI Appendix*, Fig. S1*C*), indicating that positioned loop formation is largely retained in these strains. In contrast, cells lacking both acetylations (*ECO1-AID* and *K112R-K113R*) showed a dramatic reduction in both the visibility of off-diagonal spots and the signal intensity in pile-ups [Fig. 1 *D* and *E*, *SI Appendix*, Fig. S1*C*, and (22, 23)]. These results suggest that acetylation of either K112 or K113 is sufficient to support the formation of positioned loops, while simultaneous loss of both modifications severely impairs this process, resulting in loop expansion.

Positioned loop anchors in wild-type cells correlate with the crosslinking of two CARs, most prominently between adjacent ones (+1), followed by a gradual decrease with more distal CARs until the +5 CAR (*SI Appendix*, Fig. S1*C*) (11). Robust signals for positioned loops were evident in the *K112R* and *K113R* single mutants (*SI Appendix*, Fig. S1*D*). In contrast, the distal interactions between CARs were significantly reduced in Eco1-depleted and *K112R-K113R* cells compared to wild-type cells (*SI Appendix*, Fig. S1*D*). Together, our results suggested that the accumulation of CAR-associated distal interactions required the acetylation of either K112 or K113.

The K112R and K113R single mutants were still able to form positioned loops; however, both random and positioned loops were slightly longer than in wild type (Fig. 1*C* and *SI Appendix*, Fig. S1*D*). This indicates that although each single mutant retains the ability to stop loop extrusion, both residues are required to achieve full stopping efficiency comparable to wild type.

We used chromatin immunoprecipitation (ChIP) to assess whether the differences in chromosome structure in the mutants correlated with cohesin binding to DNA. Aliquots from the same cultures were used for Micro-C XL and subjected to ChIP-seq to qualitatively evaluate the genome-wide pattern of cohesin binding or ChIP-qPCR to quantitatively assess binding at representative CARs (*SI Appendix*, Fig. S2 *A*–*C*). The ChIP-seq revealed a global pattern of cohesin binding at CARs that was indistinguishable between the mutants and the wild-type (*SI Appendix*, Fig. S2*A*). The qPCR analysis showed similar levels of cohesin binding at a representative CAR and at the centromere across all mutants (*SI Appendix*, Fig. S2*B*). Thus, the level of cohesin binding to chromosomes could not explain the differences in chromosome structure in cells with singly acetylated K112 (*K113R*) or K113 (*K112R*) compared to those with unacetylated K112 and K113 (*ECO1-AID*).

Recent studies suggest that Pds5 binding to chromatin-bound cohesin is responsible for limiting the length of distal interactions and the formation of positioned loops (22, 23). Furthermore, acetylated cohesin binds Pds5 better (22). Thus, we hypothesized that Pds5 binding to cohesin might be unaffected in the wild-type or cells with singly acetylated cohesin (*K112R* or *K113R*), leading to similar distributions of distal interactions. In contrast, Pds5 binding might be compromised in cells with unacetylated cohesin (*ECO1-AID*), leading to longer distal interactions and the loss of positioned loop anchors.

To test this model, we used ChIP-qPCR to assess Pds5 binding at CARs on chromosome arms and centromeres. The Pds5 ChIP signals serve as surrogates for Pds5 binding to cohesin, as the Pds5 ChIP signal relies on cohesin binding to CARs (33). We observed that the Pds5 signal at CARs in the *K112R* strain was indistinguishable from that of wild-type and was reduced by twofold at these sequences in *K113R* cells (*SI Appendix*, Fig. S2*C*). These results indicated that 50% binding of Pds5 to chromatin-bound cohesin was sufficient to generate the wild-type length and position of loops in *K113R* cells. The Pds5 ChIP signal in Eco1-depleted (*ECO1-AID*) cells was also reduced by twofold compared to wild-type (*SI Appendix*, Fig. S2*C*). Because this level of Pds5 binding to cohesin was similar to that of K113R cohesin, the dramatic changes in the distal interactions and loss of positioned loops for unacetylated cohesin could not be attributed to the level of Pds5 binding to chromatin-bound cohesin alone.

### Evidence That Acetylation of K112 Reduces Cohesin ATPase Activity In Vitro and In Vivo.

Several studies strongly suggested that K113 acetylation reduced Scc2/Scc4 stimulation of cohesin ATPase activity (21, 25, 26). Therefore, we wondered whether the different acetylation states of K112 might reduce cohesin ATPase activity. To compare ATPase levels across different acetylation states, we needed homogeneous populations of cohesin acetylated at K112, K113, or both. These purifications were not possible because Eco1 acetylation of K112 and K113 had not been reconstituted in vitro. In vivo, Eco1 acetylation of these residues was only partial in wild-type cells; therefore, cohesin purified from the *K113R* or *K112R* strains would only be partially acetylated at K112 or K113, respectively.

To overcome these hurdles, we expressed wild-type or mutant cohesins in *eco1∆ wpl1∆* cells, which lack acetyltransferase activity but are viable due to the absence of Wpl1 (15). In *eco1∆ wpl1∆* cells expressing wild-type cohesin, cohesin is not acetylated. In *eco1∆ wpl1∆* cells expressing the smc3-K112Q mutation (hereafter K112Q), all cohesin complexes contained only the K112 acetyl-mimic with no acetylation at K113. Similarly, expression of smc3-K113Q or the double mutant smc3-K112Q K113Q in the same background generated cohesin complexes that mimicked acetylation at K113 alone (hereafter K113Q) or at both lysines (hereafter K112Q K113Q), respectively.

**Fig. 2.**
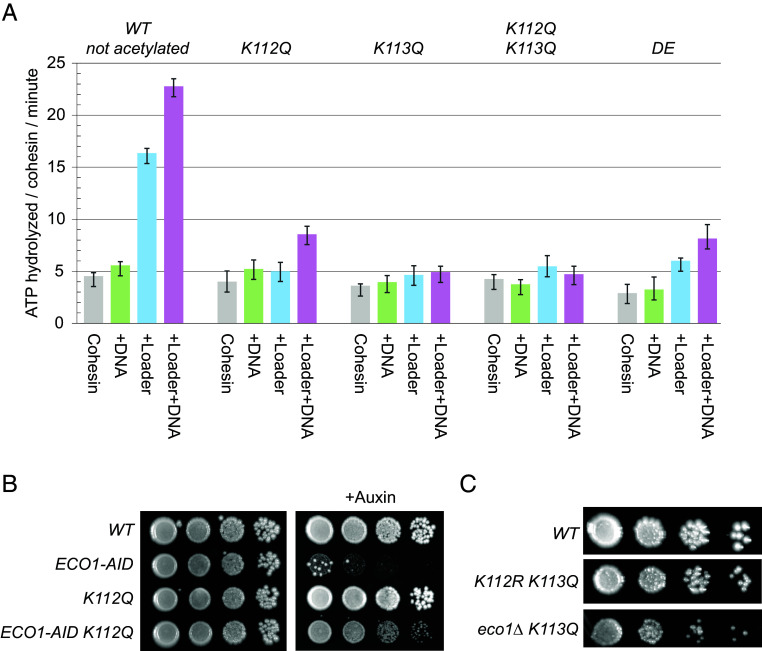
ATPase activity in wild-type and cohesin acetylation mutants. (*A*) Cohesin mutants differentially affect ATP hydrolysis rates. Wild-type and mutant cohesin complexes were purified from the acetyltransferase-defective eco1∆ wpl1∆ strains to avoid cohesin acetylation, and equal amounts were assessed for ATPase activities in the presence of DNA, with and without Scc2p/Scc4p (Loader). We tested ATP hydrolysis per minute for wild type not acetylated complex (*WT not acetylated*), single acetylation-mimic mutants smc3-K112Q (*K112Q*) and smc3-K113Q (*K113Q*), double acetylation-mimic complex (*K112Q K113Q*), and a previously characterized mutant cohesin with a low ATPase activity, smc1-D1164E (*DE*). (*B*) Cohesin smc3-K112Q acetyl-mimic mutant rescues viability in the absence of ECO1. Saturated cultures of cells with wild-type cohesin (*WT*), cells depleted for Eco1 acetyltransferase (*ECO1-AID*), cells with acetyl-mimic smc3-K112Q mutant (*K112Q*), or cells depleted for Eco1 acetyltransferase and with acetyl-mimic smc3-K112Q mutant (*ECO1-AID K112Q*), were spotted on rich media with or without auxin. (*C*) Cohesin smc3-K113Q lethality is rescued by blocking smc3-K112 acetylation. Saturated cultures of cells with wild-type cohesin (*WT*), cells with Smc3 acetyl-null mutant *K112R and* acetyl-mimic smc3-K113Q (*K112R K113Q*), or cells depleted for Eco1 acetyltransferase and with acetyl-mimic smc3-K113Q mutant (*ECO1-AID K113Q*), were spotted on rich media.

We observed that Scc2/Scc4 stimulated cohesin ATPase 4.5-fold, and this stimulation was abolished by K113Q, as previously observed (21, 26, 34). The loader stimulated ATPase only twofold for cohesin with K112Q (Fig. 2*A*). These results suggested that the loader induction of cohesin ATPase was completely blocked by K113 acetylation and partially blocked by K112 acetylation.

The reduced ATPase level of the cohesin with the K112Q mutation was similar to cohesin with the ATPase active site mutation, *smc1-D1164E* (henceforth referred to as *DE*) (Fig. 2*A*) (21, 26). If the *K112Q* mutation impacted ATPase activity in vivo as it did in vitro, the phenotypes of the *K112Q* mutant should be similar to the *DE* mutant. Like the *DE* mutant, the *K112Q* mutant had the rare ability to suppress the inviability of Eco1 depletion (Fig. 2*B*) (21). In addition, like the *DE* mutant, the *K112Q* mutant exhibited near wild-type levels of cohesion, robust DNA repair in response to camptothecin, and resistance to the mitotic stress of the microtubule inhibitor benomyl (*SI Appendix*, Fig. S3 *A* and *B*) (21, 26), These phenotypic similarities between *K112Q* and *DE* strains suggested that K112 acetylation partially represses the cohesin ATPase activity in vivo as well as in vitro.

As shown in a previous study (35), expression of the acetyl-mimic K113Q cohesin mutant alone was lethal, but viability was restored when the acetyl-null K112R mutation was added, generating the K112R K113Q double mutant (Fig. 2*C*). This suppression suggested that blocking acetylation at K112 counteracted the negative effect of the K113Q mutation, which mimicked acetylation. If suppression depended solely on blocking K112 acetylation, then deleting *ECO1*, the acetyltransferase for K112, should also restore viability to the K113Q strain, which was confirmed experimentally (Fig. 2*C*). One model proposed that in the K113Q mutant, additional acetylation at K112 further reduced cohesin ATPase activity to lethal levels, and that blocking this acetylation with K112R or ECO1 deletion prevented further inhibition and rescued growth. However, this explanation was likely excluded because cohesin complexes with K112Q K113Q double mutant and K113Q alone showed the same ATPase activity (Fig. 2*A*). Thus, the rescue of the K113Q mutant by K112R indicates that K112 acetylation contributes to cohesin regulation through a mechanism beyond simply modulating ATPase activity.

### Impact of Altering Cohesin ATPase Activity on Chromosome Structure.

**Fig. 3.**
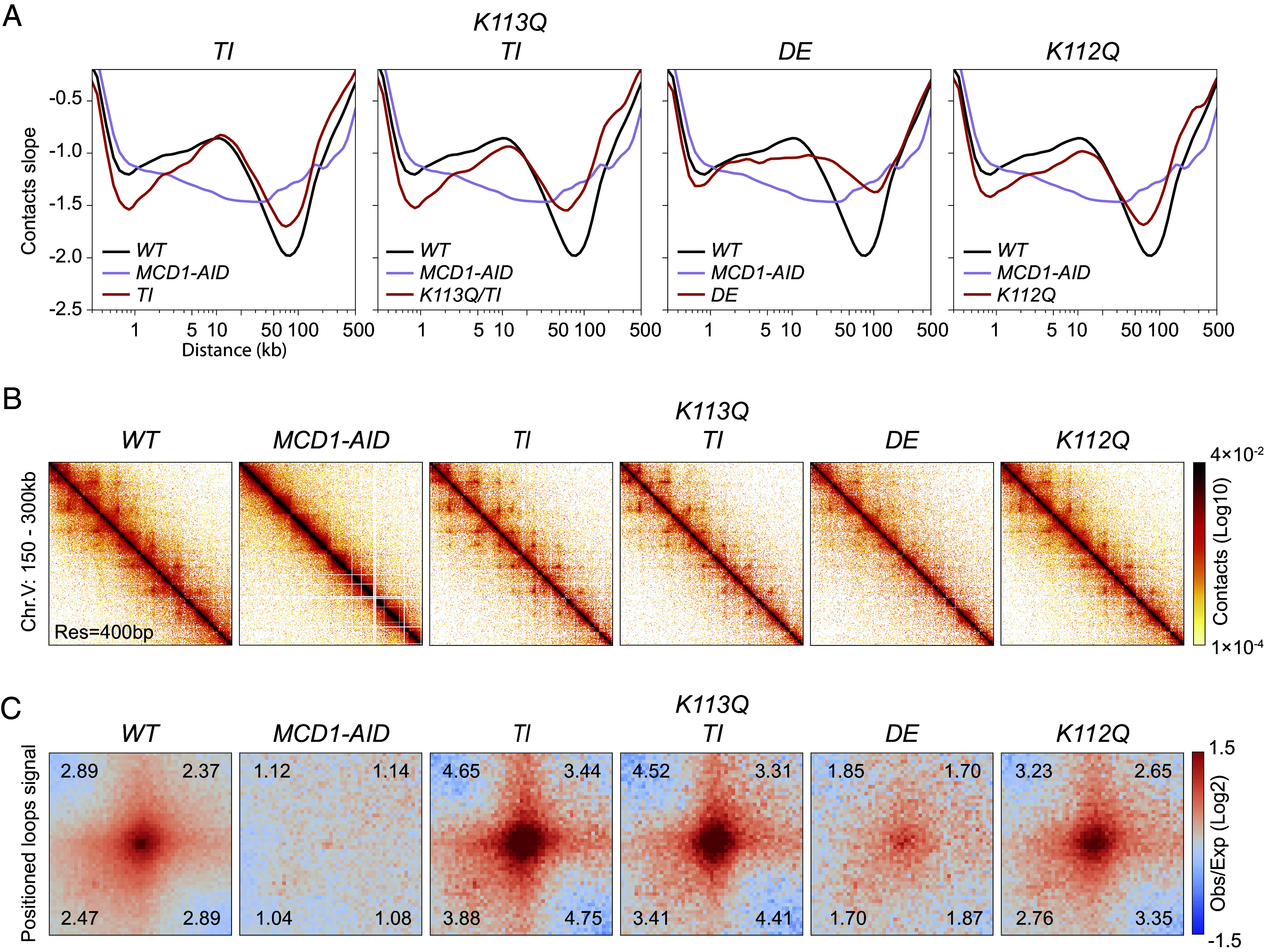
Chromosome structure in wild-type and cohesin ATPase mutants. (*A*) Chromosome contacts in cohesin ATPase mutants. Micro-C XL analysis of chromosome interactions in mitotically arrested wild-type cells (*WT*), mutants with elevated ATPase smc1-T1117I (*TI*) and smc3-K113Q combined with smc1-T1117I (*K113Q TI*), and mutants with reduced ATPase smc1-D1164E (*DE*) and smc3-K112Q (*K112Q*). Derivative slopes of the contact frequency curves (shown in *SI Appendix*, Fig. S4*A*) plotted against genomic distance are depicted in black for *WT,* mauve for *MCD1-AID*, and red for ATPase mutants. The (*B*) Contact maps in cohesin ATPase mutants. Micro-C XL contact maps at 400 bp resolution over an arm region at chromosome V 150 to 300 kb for *WT*, *MCD1-AID*, high ATPase *TI* and *K113Q TI*, and low ATPase *DE* and *K112Q* strains listed in *A*. (*C*) Genome-wide signal for positioned loops at CARs in cohesin ATPase mutants. Piled-up heatmap of the ±5 kb regions centered at CARs for *WT*, *MCD1-AID*, high ATPase *TI* and *K113Q TI*, and low ATPase *DE* and *K112Q* strains listed in *A*. Numbers in the corners represent the fold-change of the signal enrichment of the center pixel over the indicated corner pixels.

We performed Micro-C XL on a panel of mutants that alter cohesin ATPase activity to assess its impact on chromosome structure. This panel included cohesin with increased ATPase activity (26). The smc1-T1117I subunit (henceforth referred to as TI) had enhanced loader-induced ATPase activity. Cohesin with the smc3-K113Q and smc1-T1117I subunits (henceforth referred to as K113Q TI) exhibited constitutively high ATPase activity even in the absence of the loader or DNA. The derivative slopes, decay curves, contact maps, and pile-ups revealed that the length distributions and loop positioning for these strains were similar but not identical to those of wild-type (Fig. 3 and *SI Appendix*, Fig. S4). These mutants had a reduced proportion of the small loops (2 to 10 kb) in the decay curves (Fig. 3*A* and *SI Appendix*, Fig. S4*A*), likely corresponding to the reduced random loops (the off-diagonal fuzz) observed in the contact maps (Fig. 3*B* and *SI Appendix*, Fig. S4*B*). In contrast, the intensity of CAR-associated positioned loops increased relative to wild-type (Fig. 3*C* and *SI Appendix*, Fig. S4*C*). These results suggested that increased ATPase activity led to the conversion of random loops to CAR-associated positioned interactions.

This panel also included cohesin with decreased ATPase (DE and K112Q mutations). The K112Q decay curve revealed a length distribution of distal interactions similar to wild type, but these interactions were observed at a lower frequency (Fig. 3*A* and *SI Appendix*, Fig. S4*A*). The CAR-associated, positioned loop interactions were indistinguishable from the wild type (Fig. 3 *B* and *C* and *SI Appendix*, Fig. S4 *B* and *C*). These results suggested that cohesin with significantly lower induced ATPase was capable of generating a near-normal chromosome structure.

In contrast, the chromosome structure in the *DE* mutant was dramatically different from that of the wild type or the K112Q mutant. The *DE* mutant showed a reduction in interactions around the 10 kb range and a corresponding increase in distal interactions spanning 50 to 150 kb (Fig. 3*A* and *SI Appendix*, Fig. S4*A*). The contact maps and pile-ups revealed that positioned loops decreased dramatically (Fig. 3 *B* and *C* and *SI Appendix*, Fig. S4 *B* and *C*). These results suggested that in the DE mutant, looping failed to stop at CAR sites, generating much longer loops with anchors at random genomic positions. The loss of positioned loops cannot be explained by the reduced ATPase activity of DE cohesin because the K112Q cohesin had equally reduced ATPase but formed positioned loop like wild type (Figs. 2*A* and 3). Furthermore, the fact that DE cohesin could form larger loops than wild-type despite having lower ATPase activity suggested loop size in wild-type cells was not limited by cohesin’s ATPase activity.

**Fig. 4.**
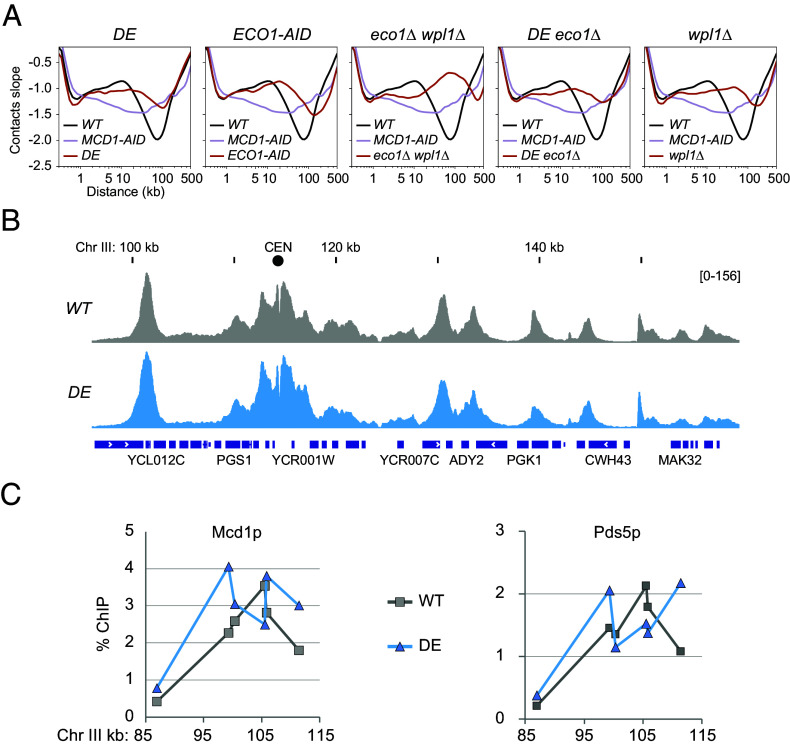
Epistasis analysis of mutants with expanded loops. (*A*) Chromosome contacts in cohesin mutants with expanded loops. Micro-C XL analysis of chromosome interactions in mitotically arrested wild-type cells (*WT*), cells depleted for cohesin (*MCD1-AID*), cells with smc1-D1164E (*DE*), cells depleted of Eco1 acetyltransferase (*ECO1-AID*), cells deleted for Eco1 and Wpl1 (*eco1∆ wpl1∆*), cells with smc1-D1164E combined with Eco1 deletion (*DE eco1∆*), cells deleted for Wpl1 (*wpl1∆*). Derivative slopes of the contact frequency curves (shown in *SI Appendix*, Fig. S5*A*) plotted against genomic distance are depicted in black for *WT,* mauve for *MCD1-AID*, and red for the indicated mutants. (*B*) Cohesin smc1-D1164E mutant binds DNA with a wild-type profile. ChIP-seq profile of mitotically arrested *WT* (gray) and *DE* (blue) cells. (*C*) Pds5 binds to the cohesin smc1-D1164E mutant on chromatin. ChIP qPCR profile of mitotically arrested *WT* (gray) and *DE* (blue) cells for Mcd1p and Pds5p on a representative CAR.

To further investigate the mechanism of DE’s longer loops, we leveraged the fact that longer distal interactions had also been observed previously upon depletion of Wpl1 or Eco1 (11, 22). Several observations suggest that these longer loops arise through distinct mechanisms in each mutant. First, analysis of contact decay curves showed that the double mutant for Eco1 and Wpl1 (*eco1∆ wpl1∆*) displayed even longer-range interactions than cells lacking only Eco1 (*Eco1-AID*) or Wpl1 (*wpl1∆*) (Fig. 4*A* and *SI Appendix*, Fig. S5*A*). Second, Eco1-depletion, unlike Wpl1-depletion, caused the loss of positioned loops (Fig. 4*A* and *SI Appendix*, Fig. S5 *B*–*D*) (11, 22). Interestingly, *DE* mutants phenocopied Eco1-depleted cells: both lacked positioned loops (*SI Appendix*, Fig. S5 *B*–*D*) and exhibited highly similar contact decay curves and derivative slopes (Fig. 4*A* and *SI Appendix*, Fig. S5*A*). In contrast, *DE* cells did not resemble the double *eco1∆ wpl1∆* mutants, which showed even more extensive looping. Together, these findings suggest an epistatic relationship between the *DE* mutation and *Eco1* depletion. Specifically, DE and Eco1 depletion act in a shared pathway that constrains loop length and supports loop positioning, distinct from the pathway affected by Wpl1.

Cohesin acetylation is known to stabilize its binding to DNA (20), and such stable binding could act as a barrier to translocating cohesin, thereby constraining loop size and promoting the formation of positioned loops. This model could explain why Eco1-depleted cells, which lack cohesin acetylation, exhibit both enlarged loops and a loss of positioned loops. Based on this reasoning, the DE mutant, which also displays long loops and reduced positioned loops, might similarly destabilize cohesin–DNA interactions. However, several lines of evidence argue against this possibility. First, ChIP-seq analysis revealed that the genome-wide pattern and intensity of cohesin binding in DE cells are comparable to those in wild-type cells (Fig. 4*B*) (21). Second, we directly tested the stability of cohesin binding at CARs. Cohesin was allowed to bind DNA during S phase, cells were then arrested in mitosis, and the Scc2 loader was inactivated to prevent new cohesin loading. We then monitored CAR-bound cohesin levels before and after loader inactivation (*SI Appendix*, Fig. S6*A*). In DE cells, cohesin levels remained unchanged at an arm CAR and decreased by ~50% at centromeres after 1 h—a stability profile indistinguishable from wild type (*SI Appendix*, Fig. S6*B*). Moreover, cohesin binding stability correlates with cohesion maintenance. The DE mutant supports near-wild-type levels of cohesion, consistent with previous reports (17, 18, 21, 26). Finally, positioned loops were not observed in eco1∆ wpl1∆ cells, where cohesin binding to chromosomes was stabilized by Wpl1 inactivation (20, 22, 23). Together, these findings argue that destabilized cohesin binding is not the shared cause of long, random loops in *DE* or Eco1-depleted cells.

Recent studies have proposed that Eco1-mediated acetylation constrains the length of cohesin-dependent loops by promoting the recruitment of Pds5 to chromatin-bound cohesin (22, 23). Based on this model, we hypothesized that the *DE* mutant could impact this mechanism by either preventing Eco1 from acetylating cohesin or altering cohesin such that acetylation can no longer enhance Pds5 binding. We tested the first possibility by looking at K113 acetylation levels using a K113ac-specific polyclonal antibody. We found that K113 acetylation was maintained in the DE mutant at levels comparable to wild type (*SI Appendix*, Fig. S6*C*), ruling out a defect in acetylation. We tested the second possibility by examining Pds5 binding to CARs in the *DE* mutant by ChIP (Fig. 4*C*). No significant difference in the Pds5 ChIP signal was observed between wild-type and *DE*, suggesting that recruitment of Pds5 to cohesin was intact in the *DE* mutant. These results indicate that the defect in the *DE* mutant likely lies downstream of Pds5 recruitment. We propose that the DE mutation alters cohesin in a way that renders it unresponsive to Pds5-dependent modulation—highlighting a previously unrecognized step in the pathway that governs the formation of positioned loops.

## Discussion

By studying a panel of cohesin mutants in budding yeast, we provide important insights into the functions and interplay of cohesin ATPase and acetylation in tethering and looping.

### The Role of Smc3 K113 Acetylation in Tethering.

Our analysis of the K113R acetylation-null mutant supports a model in which sister chromatid cohesion is established through the sequential capture of sisters (21, 36, 37). We show that the *K113R* mutant exhibits a near-wild-type distribution of cohesin binding across the genome, as well as normal-sized and positioned cohesin-dependent loops. Similar observations were reported for a cohesin hinge mutant in mammalian cells (38). Yet these mutants fail to generate cohesion. Previously, it was hypothesized that the failure of such mutants to establish cohesion might stem from unstable cohesin–DNA binding, thereby impairing cohesion maintenance (15, 39). However, subsequent studies in yeast and human cells showed that cohesin in K112R and K113R double mutants bound stably to DNA but still failed to generate cohesion (39, 40). Thus, the failure of the K113R mutant to support cohesion is not due to an inability to stably bind one sister chromatid, but rather a failure to capture or stably tether the second sister. These findings indicate that K113 acetylation is essential for the successful engagement of the second sister chromatid, and thereby, for the establishment of cohesion.

### The Role of Cohesin Tethering in Looping.

A second insight from our mutant panel study addresses the mechanism underlying chromatin loop formation in budding yeast. A recent study proposed that cohesin-dependent distal interactions may arise through a mechanism distinct from loop extrusion. In this alternative model, termed loop capture, cohesin first topologically loads onto DNA and then sequentially entraps a second DNA segment, forming a loop. Instead of active extrusion, loop formation happens passively, with transcription helping move cohesin and bring distant sites together (41). This model makes several strong predictions. First, entrapment-competent cohesin that generates cohesion and binds to wild-type sites should produce loops with wild-type length and positioning. However, we showed that DE mutant forms longer distal interactions with a near-complete loss of positioned loops, while binding to chromatin like wild type and exhibiting robust entrapment/cohesion activity (21, 26). This suggests that topological entrapment alone is not sufficient to constrain loop size or positioning in vivo.

An even stronger prediction of the loop capture model is that cohesin mutants defective in entrapping the second DNA sequence should fail both cohesion and loop formation. However, the *K113R* mutant, which binds chromatin as wild-type and is cohesion-defective, still generates loops with length and positioning similar to wild type. A similar result was reported for a cohesin hinge mutant (38). Taken together, these findings indicate that loop formation does not require the tethering activity that mediates cohesion, and instead support a model in which loops are generated through loop extrusion.

### The Role of Smc3 K112 Acetylation in ATPase Regulation and Cohesin Function.

Previous studies did not establish a functional role for the conserved acetylation of Smc3 at K112. Here, we showed that the K112 acetyl-mimic mutation reduced cohesin ATPase activity in vitro to a level intermediate between unacetylated cohesin and the K113Q acetyl-mimic. Direct measurements using cohesin homogeneously acetylated at either K112 or K113 will be necessary to confirm that glutamine substitutions faithfully recapitulate acetylation. Notably, K112Q suppressed the lethality caused by loss of Eco1-dependent cohesin acetylation. Together, these results suggested that differential acetylation of K112 and K113 generated three distinct Scc2-responsive ATPase states: fully inducible and likely loop-extruding (unacetylated), noninducible and likely cohesion-promoting (K113-acetylated), and partially inducible (K112-acetylated).

The biological significance of the partially inducible ATPase state conferred by K112 acetylation remains unclear. Previously, we showed that reduced cohesin levels supported sister chromatid cohesion but not DNA repair (42). However, altering the K112 acetylation state (K112R or K112Q) behaved like wild type in cohesion, DNA repair, and loop formation (this study). Thus, K112 acetylation is not obligatory to carry out these functions. One approach to uncover the biological function of K112 acetylation is to identify mutations that generate synthetic phenotypes in combination with the acetyl-null *K112R* mutation. This approach may reveal yet another cohesin function and provide a paradigm for studying the abundant but poorly understood posttranslational modifications of ABC ATPase proteins.

### The Role of Smc3 K112 and K113 Acetylation in Looping.

Our analysis of cohesin acetylation mutants revealed unexpected complexity in the mechanisms that govern the formation of positioned chromatin loops. We demonstrated that positioned loops formed in the *K112R* and *K113R* single mutants (this study), in contrast to their loss in the *K112R, K113R* double mutant (22). This suggests that acetylation of either K112 or K113 is necessary to support loop positioning, indicating a degree of functional overlap between the two modifications. One possible explanation is that acetylation at either site reduces cohesin ATPase activity (this study), which could stop loop extrusion, thus forming positioned loops. However, our data show that the DE mutant, which also exhibits low ATPase activity comparable to K112-acetylated cohesin (K112Q), is significantly impaired in forming positioned loops. This finding suggests that while reduced ATPase activity may be necessary, it is not sufficient for the establishment of positioned loops. Therefore, K112 and K113 acetylation must influence loop positioning through mechanisms beyond ATPase inhibition alone.

A compelling hypothesis is that K112 or K113 acetylation promotes loop positioning by enhancing cohesin’s interaction with Pds5, a factor known to bind acetylated cohesin (43), and shown to be required for positioned loop formation (11, 22, 44). However, in our study, DE cohesin was acetylated, bound Pds5 robustly, yet failed to generate positioned loops, indicating that Pds5 binding alone is also not sufficient. We propose that Pds5 must influence cohesin function or conformation. The DE mutation, which was originally selected to bypass the need for acetylation, may confer a cohesin conformation that is refractory to modulation by Pds5, thereby preventing proper loop positioning. These findings point to a more nuanced regulatory role for acetylation—beyond ATPase inhibition or cofactor recruitment in shaping cohesin’s loop-forming capacity.

### The Role of Cohesin ATPase in Looping.

Our cohesin mutant panel provided important insights into how ATPase activity influences chromosome structure. Cohesin complexes with elevated ATPase activity, such as those carrying the smc1-T1117I (TI) or smc3-K113Q with smc1-T1117I (K113Q TI) mutations, exhibited fewer randomly occurring loops and more positioned loops compared to wild type. One explanation for this pattern is that loop extrusion proceeds dynamically until cohesin encounters a boundary element that halts translocation and stabilizes a positioned loop, while random loops represent transient intermediates formed during this process. In this model, cohesin with higher ATPase activity may translocate more rapidly, spending less time in the intermediate positions. Alternatively, random loops may result from abortive looping attempts, and higher ATPase activity could enhance processivity, enabling cohesin to reach stop signals and establish stable loops more reliably. Finally, high ATPase activity may stabilize cohesin at boundary elements, thereby allowing for the accumulation of more positioned loops. These hypotheses can be tested in future in vitro loop extrusion assays.

Interestingly, we found that mutations that reduce cohesin ATPase activity did not necessarily impair the formation of chromatin loops. For example, the K112Q mutant produced distal interactions similar in length to wild type, while the DE mutant generated longer-range interactions. This raises the possibility that ATP hydrolysis may not be the rate-limiting step in the loop extrusion process, implying that ATP hydrolysis may not control how quickly or how far cohesin extrudes loops. However, there is an important caveat to this interpretation. Our experiments used Micro-C XL assays performed after mitotic arrest, meaning that cells were held in a paused state for an extended period. This pause could have allowed even slowly functioning cohesin complexes more time to extrude loops, potentially masking defects in loop extrusion speed. To determine whether ATP hydrolysis truly influences loop length and speed, it will be important to perform additional analyses—including time-resolved studies both in vivo and in vitro—to directly measure cohesin behavior under dynamic conditions.

## Materials and Methods

Details of all methods are found in *SI Appendix*, *Supplemental Methods*.

### Yeast Strains.

Yeast strains used in this study are A364A background unless otherwise specified. Genotypes are listed in *SI Appendix*, Table S1.

### Protein Extracts and Western Blotting.

Protein extracts and western blots for detecting Mcd1, Scc2-V5, and Smc3-K113 acetylation were performed as described previously (36).

### Cohesion Assay.

Cohesion was monitored at *LYS4* using the LacO-LacI system as previously described (35). Mid-M phase cells were fixed, and the number of GFP signals in each cell was scored. Cells with 2 GFP spots have defective cohesion.

### ChIP and Micro-C XL.

ChIP-qPCR, ChIP-seq, and Micro-C XL were prepared and analyzed as described previously (11, 36).

## Supplementary Material

Appendix 01 (PDF)

## Data Availability

Next-generation sequencing data have been uploaded to the National Center for Biotechnology Information (NCBI) Gene Expression Omnibus (GEO) accession number GSE310515 (45).
