# Supplementary material for: Cohesin acetylation and ATPase activity control cohesion and loop architecture through distinct mechanisms
Source: Proc Natl Acad Sci U S A. 2026 Apr 22;123(17):e2531218123. doi: 10.1073/pnas.2531218123 (PMC13123822; doi:10.1073/pnas.2531218123)
Supplement: Supplementary file 1 — Appendix 01 (PDF) [file pnas.2531218123.sapp.pdf]

Supplementary Figures, Methods and Table

Cohesin acetylation and ATPase activity control cohesion and loop architecture through distinct mechanisms

Lorenzo Costantino[1 2 4], Tiantian Ye[3 4], Kevin Boardman[1], Siheng Xiang[1], Jonathan Luo[1], Yudi Mu[3], Wenxiu Ma[3], Douglas Koshland[1]

[1]Department of Molecular and Cell Biology, University of California, Berkeley, Berkeley, California 94720, USA

[2]Research Institute of Molecular Pathology, Vienna BioCenter, Vienna 1030, Austria

[3]Department of Statistics, University of California, Riverside
900 University Ave, Riverside, CA 92521

[4]L.C. and T.Y. contributed equally to this work.

To whom correspondence may be addressed. Email: koshland@berkeley.edu (D.K.) and lorenzo.costantino@gmail.com (L.C.)

This PDF file includes:

Figures S1 to S6

Supplemental Methods

References for Supplemental Methods

Table S1

Fig.S1

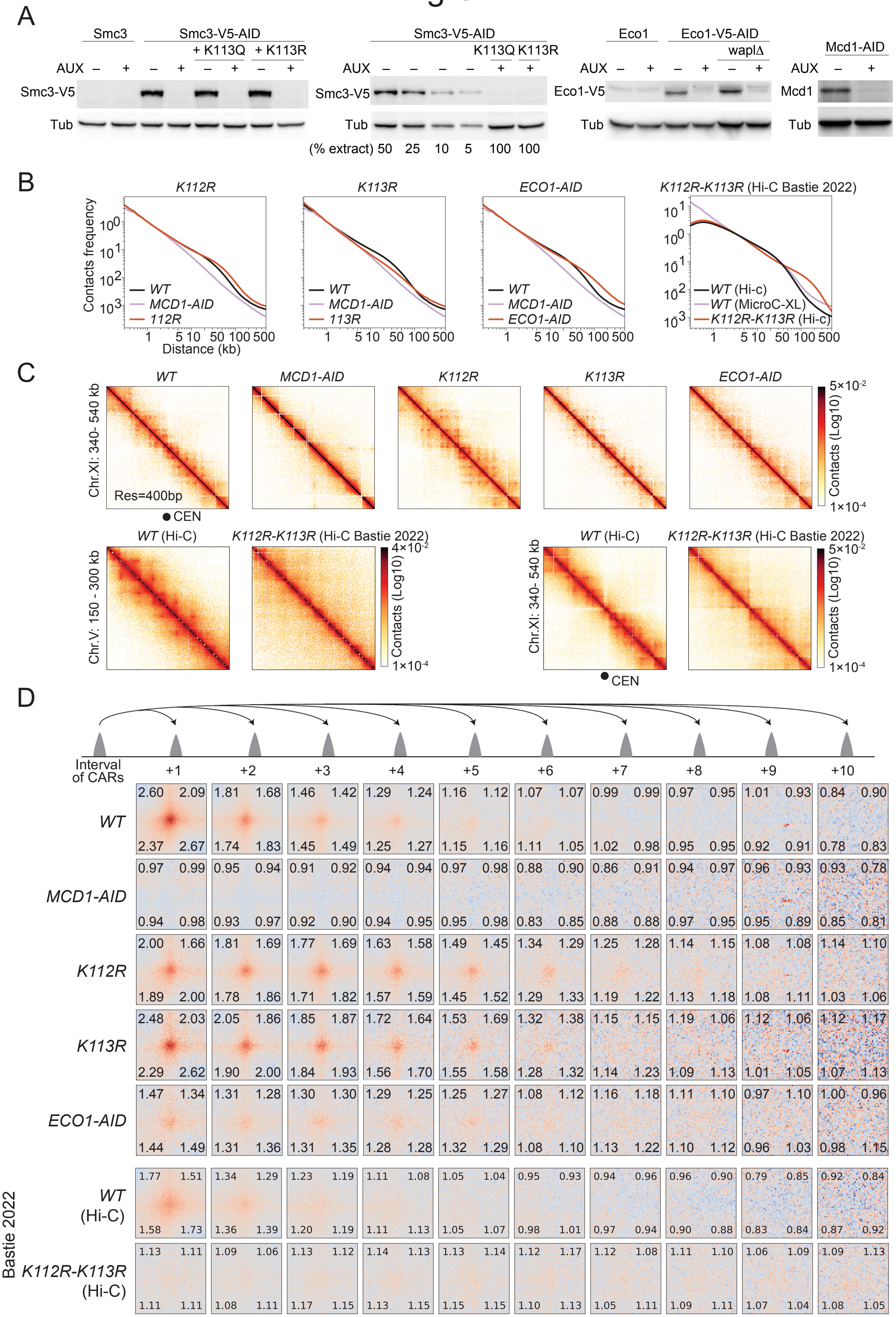
A
Smc3
Smc3-V5-AID
+ K113Q
+ K113R
AUX – + – + – + – +
Smc3-V5
Tub
Smc3-V5-AID
K113Q K113R
AUX – – – – + +
Smc3-V5
Tub
(% extract) 50 25 10 5 100 100
Eco1
Eco1-V5-AID
waplΔ
AUX – + – + – +
Eco1-V5
Tub
Mcd1-AID
AUX – +
Mcd1
Tub
B
K112R
K113R
ECO1-AID
K112R-K113R (Hi-C Bastie 2022)
Contacts frequency
Distance (kb)
1 5 10 50 100 500
WT
MCD1-AID
112R
113R
ECO1-AID
WT (Hi-c)
WT (MicroC-XL)
K112R-K113R (Hi-c)
C
WT
MCD1-AID
K112R
K113R
ECO1-AID
Chr.XI: 340- 540 kb
Res=400bp
CEN
Contacts (Log10)
5×10-2
1×10-4
WT (Hi-C)
K112R-K113R (Hi-C Bastie 2022)
Chr.V: 150 - 300 kb
4×10-2
1×10-4
Chr.XI: 340- 540 kb
D
Interval of CARs
+1 +2 +3 +4 +5 +6 +7 +8 +9 +10
WT
2.60 2.09 1.81 1.68 1.46 1.42 1.29 1.24 1.16 1.12 1.07 1.07 0.99 0.99 0.97 0.95 1.01 0.93 0.84 0.90
2.37 2.67 1.74 1.83 1.45 1.49 1.25 1.27 1.15 1.16 1.11 1.05 1.02 0.98 0.95 0.95 0.92 0.91 0.78 0.83
MCD1-AID
0.97 0.99 0.95 0.94 0.91 0.92 0.94 0.94 0.97 0.98 0.88 0.90 0.86 0.91 0.94 0.97 0.96 0.93 0.93 0.78
0.94 0.98 0.93 0.97 0.92 0.90 0.94 0.95 0.95 0.98 0.83 0.85 0.88 0.88 0.97 0.95 0.95 0.89 0.85 0.81
K112R
2.00 1.66 1.81 1.69 1.77 1.69 1.63 1.58 1.49 1.45 1.34 1.29 1.25 1.28 1.14 1.15 1.08 1.08 1.14 1.10
1.89 2.00 1.78 1.86 1.71 1.82 1.57 1.59 1.45 1.52 1.29 1.33 1.19 1.22 1.13 1.18 1.08 1.11 1.03 1.06
K113R
2.48 2.03 2.05 1.86 1.85 1.87 1.72 1.64 1.53 1.69 1.32 1.38 1.15 1.15 1.19 1.06 1.12 1.06 1.12 1.17
2.29 2.62 1.90 2.00 1.84 1.93 1.56 1.70 1.55 1.58 1.28 1.32 1.14 1.23 1.09 1.13 1.01 1.05 1.07 1.13
ECO1-AID
1.47 1.34 1.31 1.28 1.30 1.30 1.29 1.25 1.25 1.27 1.08 1.12 1.16 1.18 1.11 1.10 0.97 1.10 1.00 0.96
1.44 1.49 1.31 1.36 1.31 1.35 1.28 1.28 1.32 1.29 1.08 1.10 1.13 1.22 1.10 1.12 0.96 1.03 0.98 1.15
Bastie 2022
WT (Hi-C)
1.77 1.51 1.34 1.29 1.23 1.19 1.11 1.08 1.05 1.04 0.95 0.93 0.94 0.96 0.96 0.90 0.79 0.85 0.92 0.84
1.58 1.73 1.36 1.39 1.20 1.19 1.11 1.13 1.05 1.07 0.98 1.01 0.97 0.94 0.90 0.88 0.83 0.84 0.87 0.92
K112R-K113R (Hi-C)
1.13 1.11 1.09 1.06 1.13 1.12 1.14 1.13 1.13 1.14 1.12 1.17 1.12 1.08 1.11 1.10 1.06 1.09 1.09 1.13
1.11 1.11 1.08 1.11 1.17 1.15 1.13 1.15 1.15 1.15 1.10 1.13 1.05 1.11 1.09 1.11 1.07 1.04 1.08 1.05

Fig. S1. Chromosome structure in wild-type and cohesin acetylation mutants

(A) AID-tagged proteins are efficiently depleted upon auxin addition. Western blot analysis shows depletion of Smc3-V5-AID following auxin addition, with tubulin used as a loading control. Quantification indicates that Smc3-V5-AID levels decrease to below 5% (second blot). The depletion of Eco1-V5-AID and Mcd1-AID upon auxin addition is shown in the third and fourth blots, respectively.

(B) Chromosome contacts in cohesin acetylation mutants. Micro-C XL analysis of chromosome interactions in mitotically arrested wild-type cells (*WT*), single acetylation-deficient mutants smc3-K112R (*K112R*) and smc3-K113R (*K113R*), and cells depleted of Eco1 acetyltransferase (*ECO1-AID*) or cohesin (*MCD1-AID*) (strains genotypes in Table S1). Interactions-versus-distance decaying curve shows the normalized contact density (y-axis) against the distance between the pair of crosslinked nucleosomes from 100bp to 1Mb (x-axis). *WT* is depicted in black*, MCD1-AID* in mauve, and acetylation-mutants in red. Wild type (*WT* Hi-C) and smc3-K112R-K113R (*K112R-K113R* Hi-C) from Hi-C experiments were also plotted (Bastié et al. 2022).

(C) Contact maps in cohesin acetylation mutants over a centromere. Micro-C XL contact maps at 400bp resolution over the centromeric region at chromosome XI 340-540kb for the *WT*, *MCD1-AID*, *K112R*, *K113R*, and *ECO1-AID* strains listed in A. The centromere position is depicted with a black circle. Wild type (*WT* Hi-C) and smc3-K112R-K113R (*K112R-K113R* Hi-C) from Hi-C experiments were also plotted (Bastié et al. 2022).

(D) Genome-wide signal for positioned loops at different CAR intervals in cohesin acetylation mutants. Piled-up heatmap of the ±5kb regions centered at different intervals of CARs from +1 to +10 for the *WT*, *MCD1-AID*, *K112R*, *K113R*, and *ECO1-AID* strains listed in A. Numbers in the corners represent the fold-change of the signal enrichment of the center pixel over the indicated corner pixels. Wild type (*WT* Hi-C) and smc3-K112R-K113R (*K112R-K113R* Hi-C) from Hi-C experiments were also plotted (Bastié et al. 2022).

Fig.S2

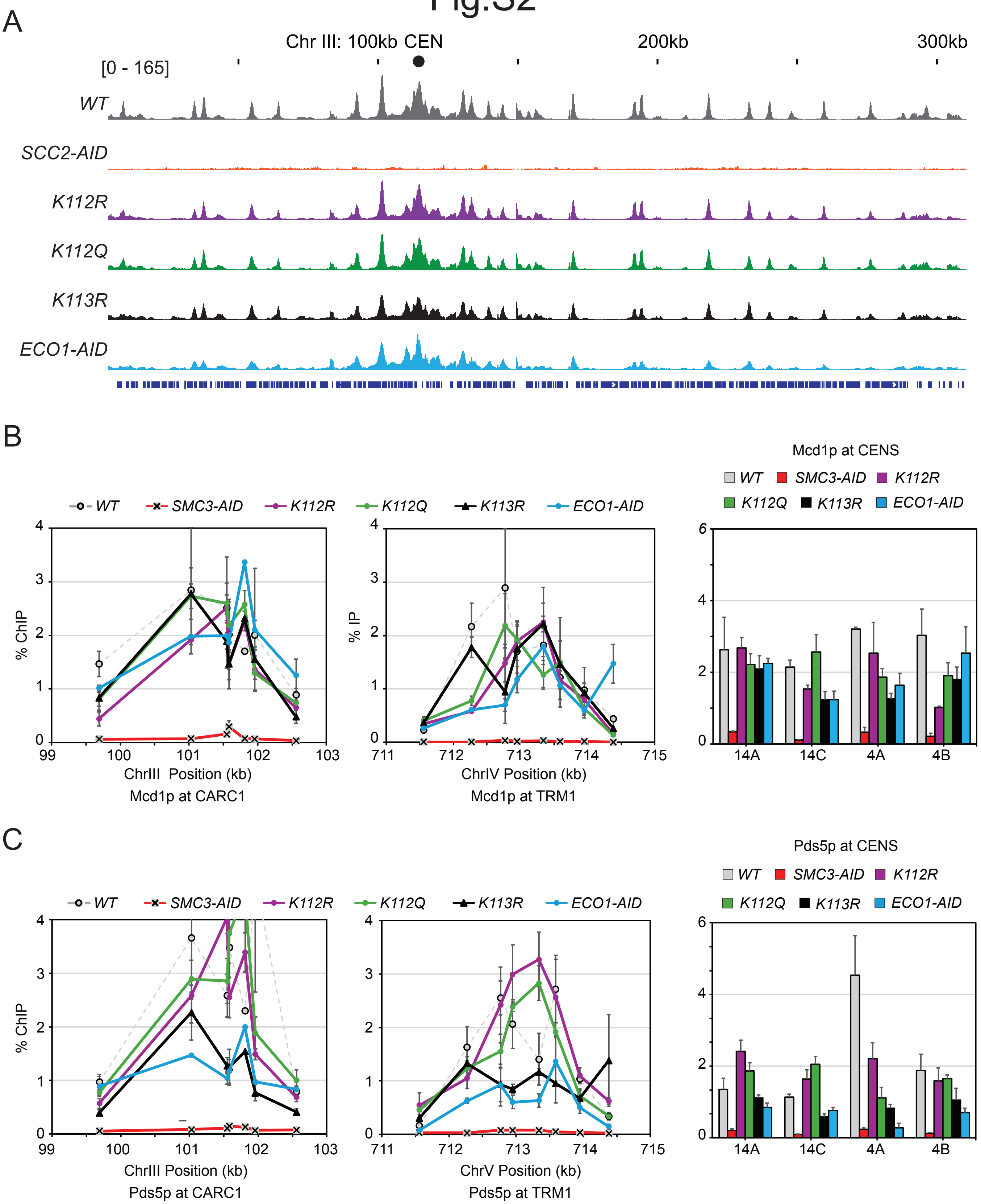
A
Chr III: 100kb CEN
200kb
300kb
[0 - 165]
WT
SCC2-AID
K112R
K112Q
K113R
ECO1-AID
B
WT
SMC3-AID
K112R
K112Q
K113R
ECO1-AID
% ChIP
ChrIII Position (kb)
Mcd1p at CARC1
% IP
ChrIV Position (kb)
Mcd1p at TRM1
Mcd1p at CENS
14A
14C
4A
4B
C
% ChIP
ChrIII Position (kb)
Pds5p at CARC1
ChrV Position (kb)
Pds5p at TRM1
Pds5p at CENS

Fig. S2. Cohesin acetylation mutants bind chromatin as wild type.

(A) Cohesin acetylation mutants bind chromatin as wild type by ChIP-seq. ChIP-seq profile of mitotically arrested wild-type cells (*WT*), cells depleted for Scc2 loader (*SCC2-AID*), cells with acetyl-null smc3-K112R (*K112R*) mutant, cells with acetyl-mimic smc3-K112Q (*K112Q*), cells with acetyl-null smc3-K113R (*K113R*), and cells depleted of Eco1 acetyltransferase (*ECO1-AID*) (strains genotypes in Table S1).

(B) Cohesin acetylation mutants can bind CARs and centromeres by ChIP-qPCR. ChIP qPCR of mitotically arrested *WT*, *SCC2-AID*, *K112R*, *K112Q*, *K113R*, and *ECO1-AID* strains listed in A, for Mcd1 binding at two representative CARS, CARC1 (right), TRM1 (center), and indicated centromeres (left).

(C) Pds5 can bind to cohesin acetylation mutants at CARs and centromeres by ChIP-qPCR. ChIP qPCR of mitotically arrested *WT*, *SCC2-AID*, *K112R*, *K112Q*, *K113R*, and *ECO1-AID* strains listed in A, for Pds5 binding at two representative CARS, CARC1 (right), TRM1 (center), and indicated centromeres (left).

Fig.S3

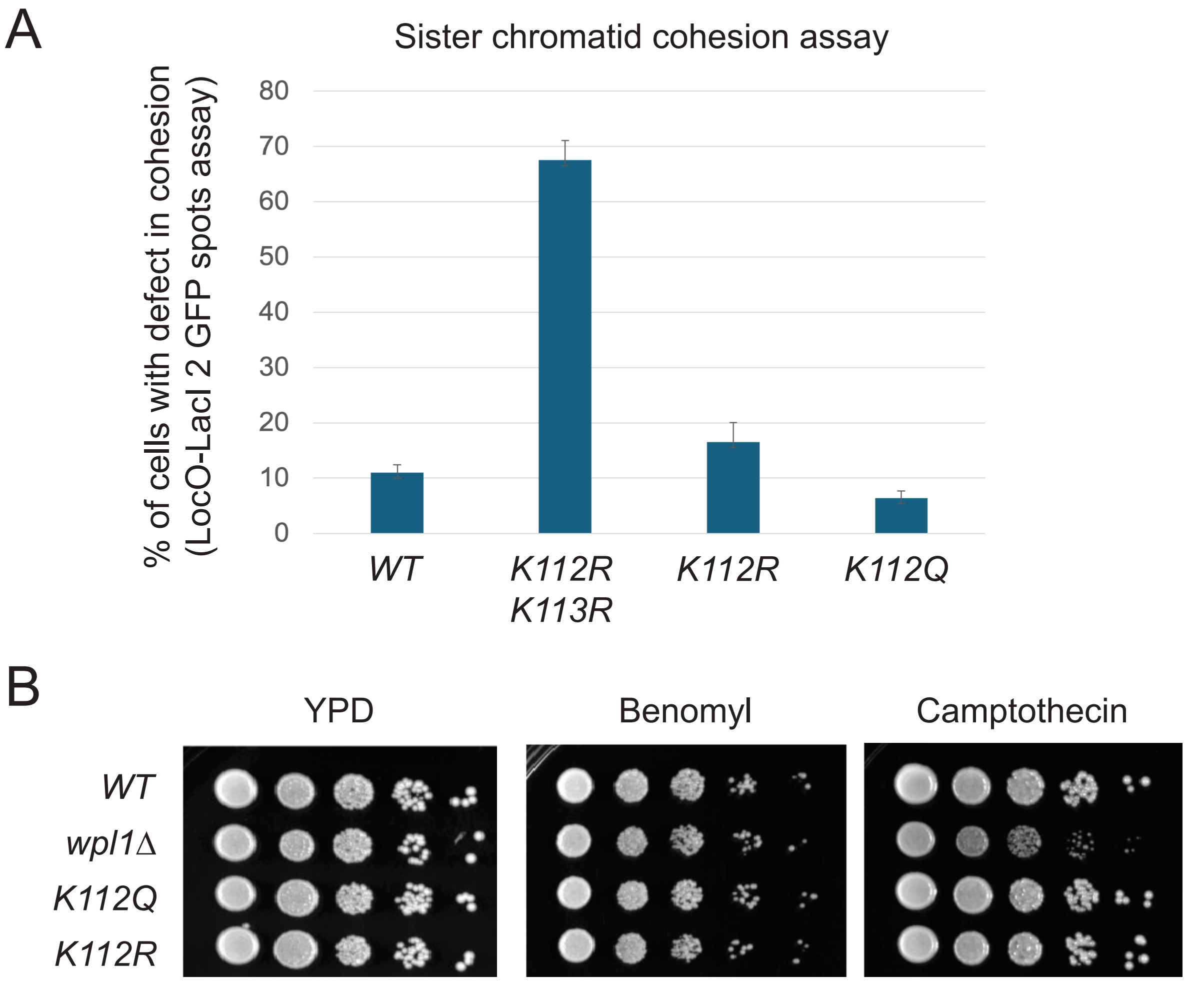

Fig. S3. Cohesin smc3-K112Q mutant in cohesion and drug sensitivity.

(A) Cohesin smc3-K112Q mutant presents wild-type levels of sister chromatid cohesion. Mitotically arrested cells were scored for cohesion defect (separated 2 GFP spots) for wild-type (*WT*), cells with smc3-K112R and smc3-K113R (*K112R K113R*), cells with smc3-K112R (*K112R*), and cells with smc3-K112Q (*K112Q*) (strains genotypes in Table S1).

(B) Cohesin smc3-K112Q mutant shows robust DNA repair and resistance to mitotic stress. Saturated cultures of cells with wild-type cohesin (*WT*), cells deleted for Wpl1 (*wpl1Δ*), cells with smc3-K112Q (*K112Q*), and cells with smc3-K112R (*K112R*) (strains genotypes in Table S1) were spotted on rich media with or without benomyl or camptothecin.

Fig.S4

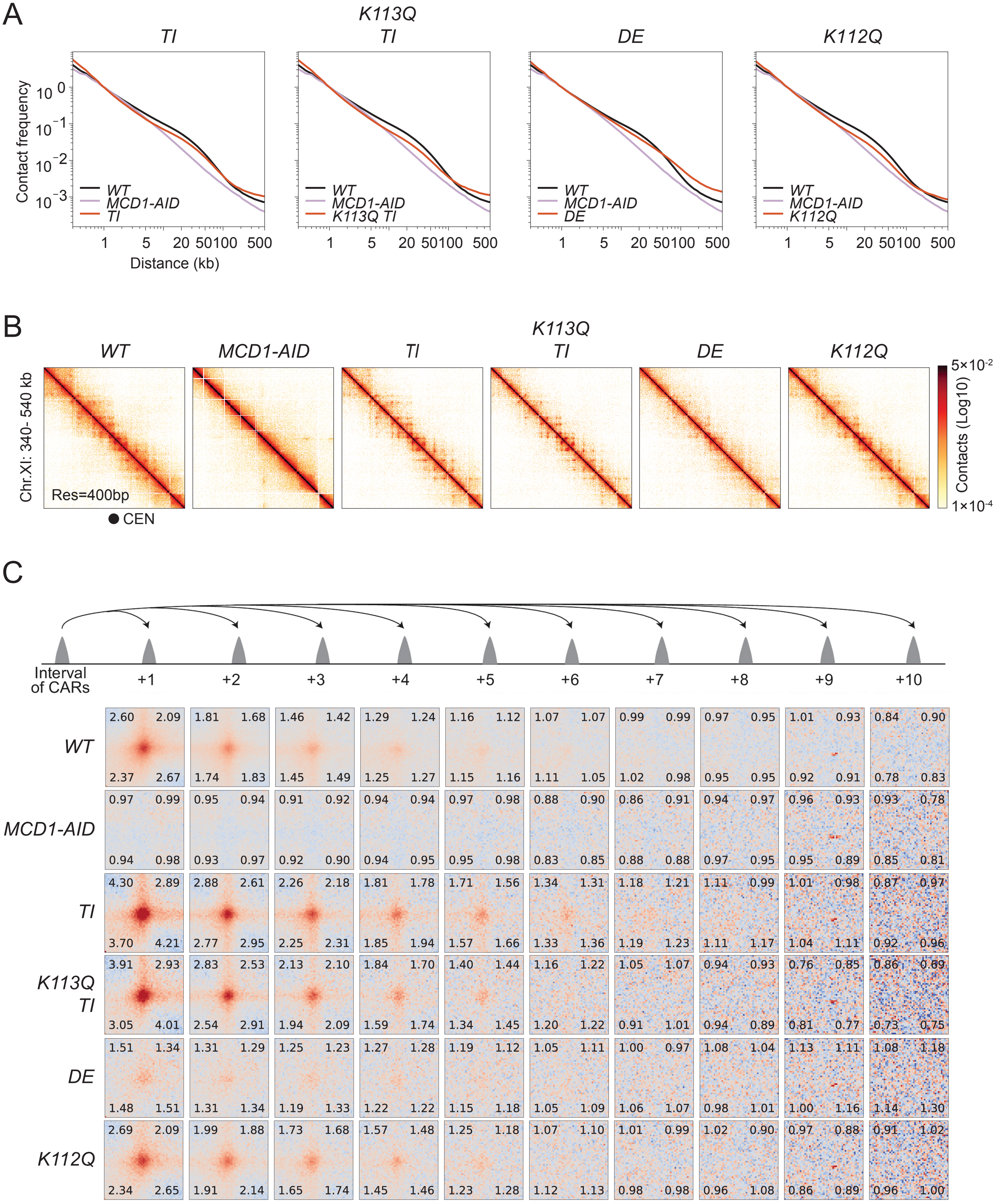
A
K113Q
TI
TI
DE
K112Q
Contact frequency
10^{0}
10^{-1}
10^{-2}
10^{-3}
WT
MCD1-AID
TI
K113Q TI
DE
K112Q
1 5 20 50100 500
Distance (kb)
B
WT
MCD1-AID
TI
K113Q TI
DE
K112Q
Chr.XI: 340- 540 kb
Res=400bp
CEN
5×10^{-2}
Contacts (Log10)
1×10^{-4}
C
Interval of CARs
+1 +2 +3 +4 +5 +6 +7 +8 +9 +10
WT
2.60 2.09 2.37 2.67 | 1.81 1.68 1.74 1.83 | 1.46 1.42 1.45 1.49 | 1.29 1.24 1.25 1.27 | 1.16 1.12 1.15 1.16 | 1.07 1.07 1.11 1.05 | 0.99 0.99 1.02 0.98 | 0.97 0.95 0.95 0.95 | 1.01 0.93 0.92 0.91 | 0.84 0.90 0.78 0.83
MCD1-AID
0.97 0.99 0.94 0.98 | 0.95 0.94 0.93 0.97 | 0.91 0.92 0.92 0.90 | 0.94 0.94 0.94 0.95 | 0.97 0.98 0.95 0.98 | 0.88 0.90 0.83 0.85 | 0.86 0.91 0.88 0.88 | 0.94 0.97 0.97 0.95 | 0.96 0.93 0.95 0.89 | 0.93 0.78 0.85 0.81
TI
4.30 2.89 3.70 4.21 | 2.88 2.61 2.77 2.95 | 2.26 2.18 2.25 2.31 | 1.81 1.78 1.85 1.94 | 1.71 1.56 1.57 1.66 | 1.34 1.31 1.33 1.36 | 1.18 1.21 1.19 1.23 | 1.11 0.99 1.11 1.17 | 1.01 0.98 1.04 1.11 | 0.87 0.97 0.92 0.96
K113Q TI
3.91 2.93 3.05 4.01 | 2.83 2.53 2.54 2.91 | 2.13 2.10 1.94 2.09 | 1.84 1.70 1.59 1.74 | 1.40 1.44 1.34 1.45 | 1.16 1.22 1.20 1.22 | 1.05 1.07 0.91 1.01 | 0.94 0.93 0.94 0.89 | 0.76 0.85 0.81 0.77 | 0.86 0.69 0.73 0.75
DE
1.51 1.34 1.48 1.51 | 1.31 1.29 1.31 1.34 | 1.25 1.23 1.19 1.33 | 1.27 1.28 1.22 1.22 | 1.19 1.12 1.15 1.18 | 1.05 1.11 1.05 1.09 | 1.00 0.97 1.06 1.07 | 1.08 1.04 0.98 1.01 | 1.13 1.11 1.00 1.16 | 1.08 1.18 1.14 1.30
K112Q
2.69 2.09 2.34 2.65 | 1.99 1.88 1.91 2.14 | 1.73 1.68 1.65 1.74 | 1.57 1.48 1.45 1.46 | 1.25 1.18 1.23 1.28 | 1.07 1.10 1.12 1.13 | 1.01 0.99 0.98 0.98 | 1.02 0.90 0.96 1.08 | 0.97 0.88 0.86 0.89 | 0.91 1.02 0.96 1.00

Fig. S4. Chromosome structure in wild-type and cohesin ATPase mutants.

(A) Chromosome contacts in cohesin ATPase mutants. Micro-C XL analysis of chromosome interactions in mitotically arrested wild-type cells (*WT*), mutants with elevated ATPase as smc1-T1117I (*TI*) and smc3-K113Q combined with smc1-T1117I (*K113Q TI)*, and mutants with reduced ATPase as smc1-D1164E (*DE*) and smc3-K112Q (*K112Q*) (strains genotypes in Table S1). Interactions-versus-distance decaying curve shows the normalized contact density (y-axis) against the distance between the pair of crosslinked nucleosomes from 100bp to 1Mb (x-axis). *WT* is depicted in black*, MCD1-AID* in mauve, and ATPase mutants in red.

(B) Contact maps in cohesin ATPase mutants over a centromere. Micro-C XL contact maps at 400bp resolution over the centromeric region at chromosome XI 340-540kb for the *WT*, *MCD1-AID*, high ATPase *TI* and *K113Q TI*, low ATPase *DE* and *K112Q* strains listed in A. The centromere position is depicted with a black circle.

(C) Genome-wide signal for positioned loops at different CAR intervals in cohesin ATPase mutants. Piled-up heatmap of the ±5kb regions centered at different intervals of CARs from +1 to +10 for the *WT*, *MCD1-AID*, high ATPase *TI* and *K113Q TI*, low ATPase *DE* and *K112Q* strains listed in A. Numbers in the corners represent the fold-change of the signal enrichment of the center pixel over the indicated corner pixels.

Fig.S5

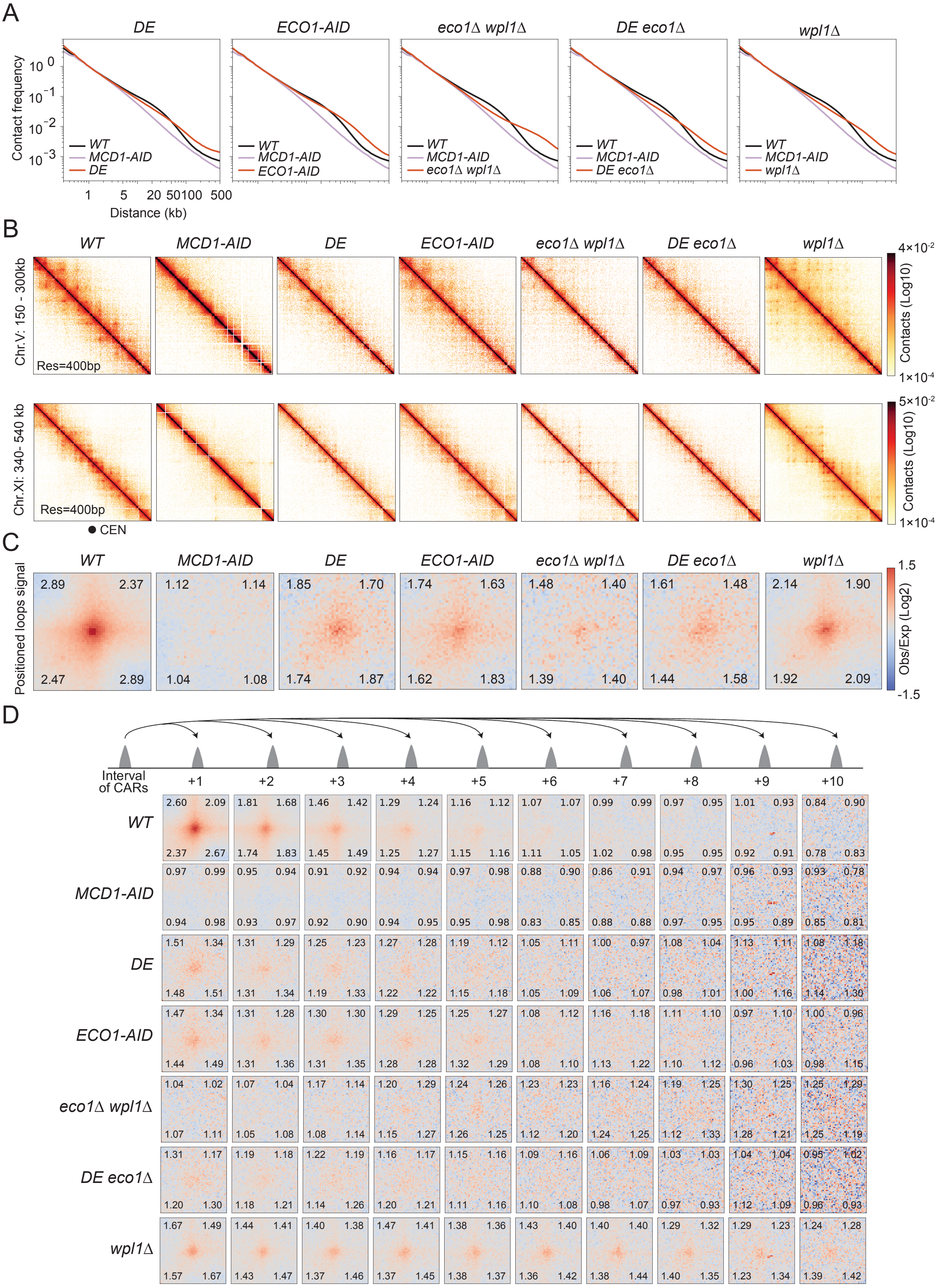
A
DE
ECO1-AID
eco1Δ wpl1Δ
DE eco1Δ
wpl1Δ
Contact frequency
10 0
10 −1
10 −2
10 −3
WT
MCD1-AID
DE
ECO1-AID
eco1Δ wpl1Δ
DE eco1Δ
wpl1Δ
1
5
20 50100
500
Distance (kb)
B
WT
MCD1-AID
DE
ECO1-AID
eco1Δ wpl1Δ
DE eco1Δ
wpl1Δ
Chr.V: 150 - 300kb
Res=400bp
4×10-2
Contacts (Log10)
1×10-4
Chr.XI: 340- 540 kb
Res=400bp
5×10-2
Contacts (Log10)
1×10-4
CEN
C
Positioned loops signal
WT 2.89 2.37 2.47 2.89
MCD1-AID 1.12 1.14 1.04 1.08
DE 1.85 1.70 1.74 1.87
ECO1-AID 1.74 1.63 1.62 1.83
eco1Δ wpl1Δ 1.48 1.40 1.39 1.40
DE eco1Δ 1.61 1.48 1.44 1.58
wpl1Δ 2.14 1.90 1.92 2.09
1.5
Obs/Exp (Log2)
-1.5
D
Interval of CARs
+1
+2
+3
+4
+5
+6
+7
+8
+9
+10
WT
MCD1-AID
DE
ECO1-AID
eco1Δ wpl1Δ
DE eco1Δ
wpl1Δ

Fig. S5. Chromosome structure of mutants with expanded loops.

(A) Chromosome contacts in mutants with expanded loops. MiMicro-C XL analysis of chromosome interactions in mitotically arrested wild-type cells (*WT*), cells depleted for cohesin (*MCD1-AID*), cells with smc1-D1164E (*DE*), cells depleted of Eco1 acetyltransferase (*ECO1-AID*), cells deleted for Eco1 and Wpl1 (*eco1∆ wpl1∆*), cells with smc1-D1164E combined with Eco1 deletion (*DE eco1∆*), cells deleted for Wpl1 (*wpl1∆*) (strains genotypes in Table S1). Interactions-versus-distance decaying curve shows the normalized contact density (y-axis) against the distance between the pair of crosslinked nucleosomes from 100bp to 1Mb (x-axis). *WT* is depicted in black*, MCD1-AID* in mauve, and mutants in red.

(B) Contact maps in mutants with expanded loops over a chromosome arm and a centromere. Micro-C XL contact maps at 400bp resolution over an arm region at chromosome V 150-300kb (top), and over the centromeric region at chromosome XI 340-540kb (bottom) for the *WT*, *MCD1-AID*, *DE, ECO1-AID, eco1∆ wpl1∆, DE eco1∆, wpl1∆* strains listed in A. The centromere position is depicted with a black circle.

(C) Genome-wide signal for positioned loops at CARs in mutants with expanded loops. Piled-up heatmap of the ±5kb regions centered at CARs for *WT*, *MCD1-AID*, *DE, ECO1-AID, eco1∆ wpl1∆, DE eco1∆, wpl1∆* strains listed in A. Numbers in the corners represent the fold-change of the signal enrichment of the center pixel over the indicated corner pixels.

(D) Genome-wide signal for positioned loops at different CAR intervals in mutants with expanded loops. Piled-up heatmap of the ±5kb regions centered at different intervals of CARs from +1 to +10 for the *WT*, *MCD1-AID*, *DE, ECO1-AID, eco1∆ wpl1∆, DE eco1∆, wpl1∆* strains listed in A. Numbers in the corners represent the fold-change of the signal enrichment of the center pixel over the indicated corner pixels.

Fig.S6

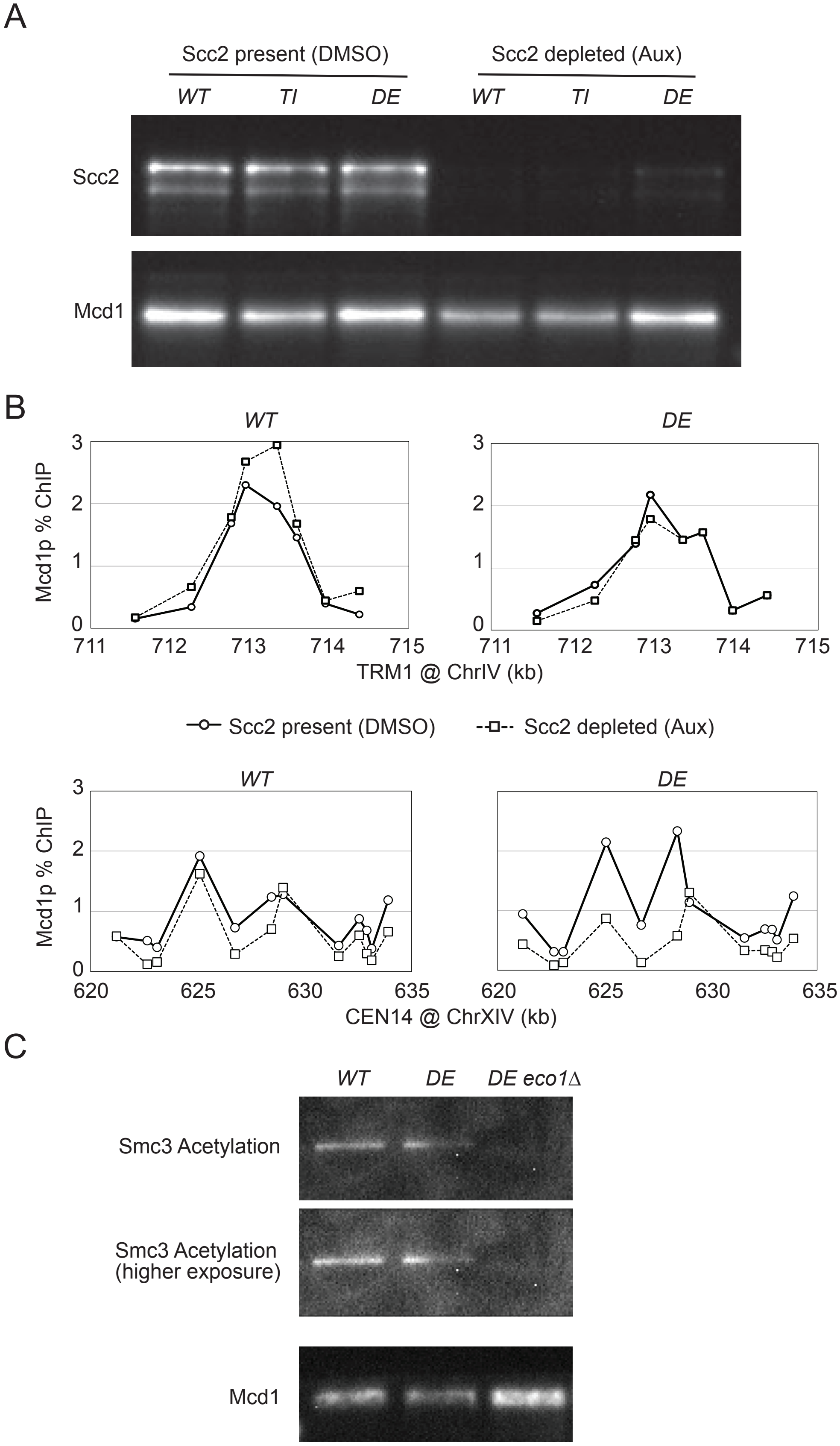
A
Scc2 present (DMSO)
Scc2 depleted (Aux)
WT
TI
DE
WT
TI
DE
Scc2
Mcd1
B
WT
DE
Mcd1p % ChIP
0
1
2
3
711
712
713
714
715
TRM1 @ ChrIV (kb)
Scc2 present (DMSO)
Scc2 depleted (Aux)
WT
DE
Mcd1p % ChIP
620
625
630
635
CEN14 @ ChrXIV (kb)
C
WT
DE
DE eco1Δ
Smc3 Acetylation
Smc3 Acetylation
(higher exposure)
Mcd1

Fig. S6. Cohesin smc1-D1164E mutant does not affect cohesin binding stability or acetylation.
(A) Scc2p is depleted efficiently by auxin. Western blot analysis for Scc2 and Mcd1 using extracts from *WT* cells, *TI* cells, and *DE* cells treated with auxin (Aux) or control (DMSO).
(B) Cohesin stability on chromatin is not affected by the smc1-D1164E mutation. ChIP qPCR for Mcd1p was performed on wild-type (*WT*) and smc1-D1164E (*DE*) cells, arrested in mitosis and subsequently depleted for Scc2 with auxin (Aux, circles with solid line) or control (DMSO, squares with dotted line). A representative CAR (TRM1 on top) and a centromere (CEN14 on bottom) were probed.
(C) Smc3 acetylation levels are not affected by smc1-D1164E mutant. Western blot analysis for Smc3 acetylation and Mcd1 using extracts from *WT* cells, *DE* cells, and *DE eco1*Δ cells.

Supplemental Methods

Yeast strains, media, and reagents.

Yeast strains used in this study are A364A background unless otherwise specified. Genotypes are listed in Supplemental Table S1. YPD media was prepared as previously described (Guacci et al., 1997). Plates containing benomyl or camptothecin (Sigma catalog # C9911) were used to assess drug sensitivity, as previously described (Guacci and Koshland 2012). Auxin (3-indoleacetic acid) (Sigma-Aldrich, St. Louis, MO) was prepared as a 1 M stock solution in DMSO and then added to the liquid media or plates at a final concentration of 500 μM or 750 μM, respectively.

Cohesin Purification Media: Low Biotin Synthetic Complete (LBSC) Media contained 1.56 g/L BSM Powder (Sunrise Science Products Cat#1387), 1.71 g/L YNB – Biotin powder (Sunrise Science Products Cat#1523), 38 mM ammonium sulfate (5 g/L), 1 nM D-biotin (Invitrogen #B20656), and 2% raffinose.

Cohesin Loader Purification Media: Low Biotin URA- Dropout Media contained 0.8g/L CSM-Ura (Sunrise Science Products), 1.71g/L YNB –Biotin powder (Sunrise Science Products Cat#1523), 38 mM ammonium sulfate (5g/L), 1 nM biotin, and 2% raffinose.

CRISPR guide plasmids and PCR-generated repair templates were made and used to insert mutations into yeast as previously described (Saxton and Rine 2019).

Preparation of mitotically arrested cells for Micro-C XL, ChIP, and cohesion assays. Asynchronous mid-log cultures were arrested in G1 by the addition of alpha factor as previously described (Guacci et al. 2019). When required, auxin was added (500 μM) to G1-arrested cells, incubated for 30 minutes while arrested in G1. G1-arrested cells were released from G1 into either YPD or YEPRG containing nocodazole and Pronase E as previously described (Guacci et al. 2019), then incubated at 30°C for 2.5h for YPD or 4h for YEPRG to arrest in mid-M phase. When required, auxin was added (500 μM) in all wash media and in resuspension media to ensure AID-tagged protein depletion.

Protein extracts and western blotting

Protein extracts and western blots for detecting Mcd1, Scc2-V5, and Smc3-K113 acetylation were performed as described previously (Eng et al. 2014; Robison et al. 2018).

Cohesion Assay

Cohesion was monitored at *LYS4* using the LacO-LacI system as previously described (Guacci and Koshland 2012). Mid-M phase cells were fixed, and the number of GFP signals in each cell was scored. Cells with 2 GFP spots have defective cohesion.

Chromatin Immunoprecipitation (ChIP) and Micro-C XL

ChIP-qPCR, ChIP-seq, and Micro-C XL were prepared and analyzed as described previously (Eng et al. 2014; Costantino et al. 2020). NGS files are deposited in GEO repository GSE310515.

Purification of cohesins and ATPase assay

Purifications of loader (SX305) and cohesin from wild type (KB58A) and mutants (SX364, KB140A, SX365, and SX316) were performed as described previously (Boardman et al. 2023). ATPase activity of cohesin was measured using EnzChek Phosphate Assay Kit with purified recombinant proteins depleted of free

phosphate using Inorganic Phosphate Binding Resin (Abcam: ab270547). Reactions were assembled with 10nM cohesin, 15nM Scc3, alone or with 65nM Scc2/4, 0.1mg/ml BSA, and 450nM 60-mer dsDNA in ATPase reaction buffer (25mM HEPES pH7.5, 20% glycerol, 50 mM NaCl, 1 mM $MgCl_2$); reactions were initiated with the addition of ATP to a final concentration of 1mM. Spectrophotometric measurements at 360 nm were taken every 1 min for 2h at room temperature. ATPase activities were calculated by linear regression of the raw data using GraphPad Prism software.

Processing Micro-C XL reads to contact matrices

Each Micro-C XL fastq raw file was processed into contact read pairs using HiC-Pro version 3.1.0 pipeline (https://github.com/nservant/HiC-Pro, Servant et al., 2015). Reads were aligned to the yeast *sacCer3* genome using Bowtie2 version 2.4.4 (Langmead and Salzberg, 2012) with '--very-sensitive-local' option. Singleton reads, multi-mapped reads, and PCR duplicate read pairs were discarded. Aligned read pairs with genomic distances shorter than 200 bp were also filtered out. The output valid contact pairs were binned at multiple resolutions and converted to contact matrices in both .cool format using cooler (https://github.com/open2c/cooler, Abdennur and Mirny, 2020) and .hic format using Juicer (https://github.com/aidenlab/juicer, Durand et al., 2016) for downstream analyses. Contact frequency matrices in .cool format were normalized using IC (iterative correction) (Imakaev et al., 2012) via the *cooler balance* command, and matrices .hic format were normalized by KR (Knight-Ruiz) balancing (Knight and Ruiz, 2013) using Juicer. The MCD1-AID mutant contact matrices in both .cool and .hic format were downloaded from GEO (GSE151553, Costantino et. al, 2020). Hi-C datasets for WT (CH112, CH233, CH283) and *Smc3 RR*, *Smc3-AID* (CH212, CH287) (Bastié et al., 2022) were downloaded from the SRA database (PRJNA715343). Hi-C fastq files were processed in a similar way as Micro-C XL data, with an additional step to generate restriction fragments after DpnII + HinfI restriction enzymes digestion. Only aligned reads that could be assigned to a restriction fragment were retained. Replicates were merged and normalized into single contact matrices in both .cool and .hic format. Heatmaps of contact matrices were plotted at 400 bp resolution using matplotlib version 3.8.3 (Hunter, 2007).

Contact frequency versus genomic distance decay curves analysis

We used intra-chromosomal 'UNI' orientation contact read pairs binned at 100 bp resolution to calculate the average contact frequency between pairs separated by the same genomic distance range (decay curves), using the *expected* module from cooltools version 0.6.1 (https://github.com/open2c/cooltools, Open2C, et al., 2024). The 'UNI' orientation is defined as aligned read pairs mapping to the same strand direction (either +/+ or -/-) (Hsieh et al., 2015). Filtered pairs were then converted into contact matrices in .cool format. The average contact frequencies at each genomic distance (matrix diagonals) were calculated independently for each chromosome using the *diagsum_symm* function from cooltools. To account for the sparsity of contacts at larger genomic distances, the data were grouped into logarithmically spaced bins (20 bins per order of magnitude) using *logbin_expected* function from cooltools. Genome-wide averages of log-binned average contact frequencies and decay slopes were then computed by combining chromosome-level values with *combine_binned_expected* function from cooltools. Decay curves, slopes, and slope differences (Δslope) were plotted using matplotlib. Micro-C XL and Hi-C decay curves were normalized at 1 kb and 3.2 kb distances, respectively.

Chromatin loops and pileup analysis

Loops were identified using the HiCCUPS algorithm (Rao et al., 2014) implemented in Juicer. We applied the algorithm to KR-normalized contact matrices in .hic format at 500 bp resolution, and the results were filtered at 1% false discovery rate. Loops were called at two settings, then merged: peak/window width (6,12) and (8,16). Pixels within 2500 bp of each other were merged. The calling options were: hiccups -m 4096 -k KR -r 500,500 -f 0.1,0.1 -p 6,8 -i 12,16 -d 2500,2500 (Costantino et al., 2020). Loop anchors separated by distance greater than 100 kb were considered likely false positives and discarded. Genome-wide positional loop signals were quantified using aggregate peak analysis (Rao et al., 2014). Pileups were generated on IC-normalized contact matrices in .cool format at 200 bp resolution using coolpup.py version 1.1.0 (https://github.com/open2c/coolpuppy, Flyamer et al., 2020). Pileups were centered at the wild-type loop anchors with ±5 kb flanking regions. To account for distance-dependent decay effects, pileups were normalized against background contact levels using randomly shifted control regions. The calling options were: minshift=1000, maxshift=10000, flank=5000, nshifts=10. Loop enrichment was calculated as the ratio of the mean normalized center contacts (within a 5x5 window) to the mean normalized corner contacts. Pileups were plotted with matplotlib.

The same aggregate peak analysis was applied to measure target-centered loop signals, using paired MCD1 ChIP-seq peaks (CARs) instead of HiCCUPS-called loop anchors. ChIP-seq raw fastq files were downloaded from the SRA database (SRR11872088 and SRR11872089, Costantino et. al, 2020). Reads were aligned to the yeast *sacCer3* genome using Bowtie2. Peaks were called with MACS2 version 2.2.7 (Zhang et al., 2008) with default settings, and replicates were combined using IDR version 2.0.4 (https://github.com/nboley/idr, Li et al., 2011) at a threshold of 0.05. MCD1 peaks were paired by genomic intervals ranging from +1 to +10, where +1 denotes the directly adjacent peaks.

Reference for Supplemental Methods

1. Servant, N., Varoquaux, N., Lajoie, B.R., Viara, E., Chen, C.J., Vert, J.P., Heard, E., Dekker, J. and Barillot, E., 2015. HiC-Pro: an optimized and flexible pipeline for Hi-C data processing. Genome biology, 16(1), p.259.

2. Langmead, B. and Salzberg, S.L., 2012. Fast gapped-read alignment with Bowtie 2. Nature methods, 9(4), pp.357-359.

3. Abdennur, N. and Mirny, L.A., 2020. Cooler: scalable storage for Hi-C data and other genomically labeled arrays. Bioinformatics, 36(1), pp.311-316.

4. Durand, N.C., Shamim, M.S., Machol, I., Rao, S.S., Huntley, M.H., Lander, E.S. and Aiden, E.L., 2016. Juicer provides a one-click system for analyzing loop-resolution Hi-C experiments. Cell systems, 3(1), pp.95-98.

5. Imakaev, M., Fudenberg, G., McCord, R.P., Naumova, N., Goloborodko, A., Lajoie, B.R., Dekker, J. and Mirny, L.A., 2012. Iterative correction of Hi-C data reveals hallmarks of chromosome organization. Nature methods, 9(10), pp.999-1003.

6. Knight, P.A. and Ruiz, D., 2013. A fast algorithm for matrix balancing. IMA Journal of Numerical Analysis, 33(3), pp.1029-1047.

7. Costantino, L., Hsieh, T.H.S., Lamothe, R., Darzacq, X. and Koshland, D., 2020. Cohesin residency determines chromatin loop patterns. Elife, 9, p.e59889.

8. Bastié, N., Chapard, C., Dauban, L., Gadal, O., Beckouet, F. and Koszul, R., 2022. Smc3 acetylation, Pds5 and Scc2 control the translocase activity that establishes cohesin-dependent chromatin loops. Nature structural & molecular biology, 29(6), pp.575-585.

9. Hunter, J.D., 2007. Matplotlib: A 2D graphics environment. Computing in science & engineering, 9(03), pp.90-95.

10. Open2C, Abdennur, N., Abraham, S., Fudenberg, G., Flyamer, I.M., Galitsyna, A.A., Goloborodko, A., Imakaev, M., Oksuz, B.A., Venev, S.V. and Xiao, Y., 2024. Cooltools: enabling high-resolution Hi-C analysis in Python. PLOS Computational Biology, 20(5), p.e1012067

11. Hsieh, T.H.S., Weiner, A., Lajoie, B., Dekker, J., Friedman, N. and Rando, O.J., 2015. Mapping nucleosome resolution chromosome folding in yeast by micro-C. Cell, 162(1), pp.108-119.

12. Rao, S.S., Huntley, M.H., Durand, N.C., Stamenova, E.K., Bochkov, I.D., Robinson, J.T., Sanborn, A.L., Machol, I., Omer, A.D., Lander, E.S. and Aiden, E.L., 2014. A 3D map of the human genome at kilobase resolution reveals principles of chromatin looping. Cell, 159(7), pp.1665-1680.

13. Flyamer, I.M., Illingworth, R.S. and Bickmore, W.A., 2020. Coolpup. py: versatile pile-up analysis of Hi-C data. Bioinformatics, 36(10), pp.2980-2985.

14. Zhang, Y., Liu, T., Meyer, C.A., Eeckhoute, J., Johnson, D.S., Bernstein, B.E., Nusbaum, C., Myers, R.M., Brown, M., Li, W. and Liu, X.S., 2008. Model-based analysis of ChIP-Seq (MACS). Genome biology, 9(9), p.R137.

15. Li, Q., Brown, J.B., Huang, H. and Bickel, P.J., 2011. Measuring reproducibility of high-throughput experiments.

Table S1 (Yeast Strains)

Shorthand Genotype	Strain	Mating Type	Genotype
**Micro C XL**			
WT	VG3620-4C	MATa	*TIR1-CgTRP1 LacO-NAT::lys4 GFPLacI-HIS3:his3-11,15 leu2-3,112 ura3-52 bar1 GAL+*
MCD1-AID	DK5501	MATa	*MCD1-AID-KANMX6 ADH1-OsTIR1- URA3::ura3-52 lys4::LacO(DK)-NAT trp1-1 GFPLacI-HIS3: his3-11,15 bar1 leu2-3,112*
K112R	VG4119-5B	MATa	*smc3-K112R TIR1-CgTRP1 LacO-NAT::lys4 GFPLacI-HIS3:his3-11,15 leu2-3,112 ura3-52 bar1 GAL+*
K112Q	VG4118-1A	MATa	*smc3-K112Q TIR1-CgTRP1 LacO-NAT::lys4 GFPLacI-HIS3:his3-11,15 leu2-3,112 ura3-52 bar1 GAL+*
K113R	TE440	MATa	*smc3-K113R-URA3::ura3-52 SMC3-3V5-AID608 TIR1-CaTRP1::trp1-1 LacO-NAT::lys4 pHIS3-GFP-LacI-HIS3:his3- 11,15 ura3-52 bar1*
K113Q	VG3981-6B	MATa	*SMC3-K113Q-LEU2:leu2-3,112 smc3-N607-3V5-AID TIR1-CaTRP1 LacO-NAT::lys4 GFPLacI-HIS3:his3-11,15 ura3-52 bar1 GAL+*
K113Q TI	VG4010-8B	MATa	*smc3-K113Q smc1-T1117I-LEU2:leu2-3,112 smc1Δ::HPH LacO-NAT::lys4 GFPLacI-HIS3:his3-11,15 trp1-1 ura3-52 bar1 GAL+*
TI	VG4006-13A	MATa	*smc1-T1117I-LEU2:leu2-3,112 smc1Δ::HPH LacO-NAT::lys4 GFPLacI-HIS3:his3-11,15 trp1-1 ura3-52 bar1 GAL+*
wpl1Δ	VG3360-3D	MATa	*ECO1-3V5-AID2-G418 TIR1-CaTRP1 LacO-NAT::lys4 leu2-3,112 GAL+ pHIS3-GFPLacI-HIS3:his3-11,15 bar1 ura3-52*
ECO1-AID wpl1Δ	VG3687-2A	MATa	*ECO1-3V5-AID2-G418 rad61Δ::HPH TIR1-CaTRP1 LacO(DK)-NAT::lys4 pHIS3-GFPLacI-HIS3:his3-11,15 bar1 leu2-3,112 ura3-52 GAL+*

ECO1-AID	VG3633-2D	MATa	*ECO1-3V5-AID2-G418 TIR1-CaTRP1 LacO(DK)-NAT::lys4 leu2-3,112 GAL+ GFPLacI-HIS3:his3-11,15 bar1 ura3-52*
DE	VG3581-8B	MATa	*smc1-D1164E-LEU2:leu2-3,112 trp1-1 smc1Δ::HPH LacO(DK)-NAT::lys4 bar1 GFPLacI-HIS3:his3-11,15 ura3-52 GAL+*
DE eco1Δ	VG3800-3A	MATa	*eco1Δ::G418 smc1-D1164E-LEU2:leu2-3,112 smc1Δ::HPH trp1-1 LacO-NAT::lys4 GFPLacI-HIS3:his3-11,15 ura3-52 GAL+ bar1*
**Suppression**			
K112R, K113Q	VG4023-2A	MATa	smc3-K112R,K113Q-LEU2:leu2-3,112 smc3Δ::HPH LacO-NAT::lys4 GFPLacI-TRP1:his3-11,15 trp1-1 ura3-52 bar1 GAL+
eco1Δ K113Q	KB106A	MATa	ctf7Δ::G418 smc3-K113Q LacO(DK)-NAT::lys4 pHIS3-GFPLacI-HIS3:his3-11,15 bar1 trp1-1 leu2-3,112 ura3-52 GAL+
K112Q	SX366	MATa	trp1Δ::pGPD1-TIR1-CaTRP1 lys4::LacO(DK)-NAT pHIS3-GFPLacI-HIS3:his3-11,15 smc3-K112Q ECO1-3V5-AID2-KANMX leu2-3,112 ura3-52 bar1
**Stability of cohesin DNA binding**			
SCC2-AID	VG3612-2C	MATa	scc2-3V5-AID2-G418 TIR1-CaTRP1 GFPLacI-HIS3:his3-11,15 LacO(DK)-NAT::lys4 leu2-3,112 bar1 ura3-52 GAL+
DE SCC2-AID	KB162	MATa	scc2-3V5-AID2-G418 Smc1-T1117I TIR1-CaTRP1 pHIS3-GFPLacI-HIS3:his3-11,15 LacO(DK)-NAT::lys4 leu2-3,112 bar1 ura3-52 GAL+

Cohesin purification

Loader	SX305	MATa	lys2 pep4::HIS3 ade2-1::ADE2-pGAL-GAL4 trp1Δ2 leu2-3, 112 ura3-52 ade2-1 can1-100 bar1::hisG w/[2u URA3 pGAL1,10-(Scc2-MYC-Strept)-(Scc4)]
	KB58A	MATa	ade2-1::ADE2-pGAL1,10-GAL4, SMC1-PK3 ura3::URA3-pGAL1,10-SCC3-MYC trp1::TRP1-pGAL1,10-SMC3,MCD1-3C-3xStrepII can1-100, leu2-3,112, his3, GAL, psi+ pep4D::HIS3 wpl1D::LEU eco1D::KANMX6
	SX364	MATa	ade2-1::ADE2-pGAL1,10-GAL4, SMC1-PK3 ura3::URA3-pGAL1,10-SCC3-MYC trp1::TRP1-pGAL1,10-SMC3-K112Q,MCD1-3C-3xStrepII can1-100, leu2-3,112, his3, GAL, psi+ pep4D::HIS3 wpl1D::LEU eco1D::KANMX6
	KB140A	MATa	ade2-1::ADE2-pGAL1,10-GAL4, SMC1-PK3 ura3::URA3-pGAL1,10-SCC3-MYC trp1::TRP1-pGAL1,10-SMC3-K113Q,MCD1-3C-3xStrepII can1-100, leu2-3,112, his3, GAL, psi+ pep4D::HIS3 wpl1D::LEU eco1D::KANMX6
	SX365	MATa	ade2-1::ADE2-pGAL1,10-GAL4, SMC1-PK3 ura3::URA3-pGAL1,10-SCC3-MYC trp1::TRP1-pGAL1,10-SMC3-KK112QQ,MCD1-3C-3xStrepII can1-100, leu2-3,112, his3, GAL, psi+ pep4D::HIS3 wpl1D::LEU eco1D::KANMX6
	SX316	MATa	ade2-1::ADE2-pGAL1,10-GAL4, SMC1-D1163E-PK3 ura3::URA3-pGAL1,10-SCC3-MYC trp1::TRP1-pGAL1,10-SMC3,MCD1-3C-3xStrepII can1-100, leu2-3,112, his3, GAL, psi+ pep4D::HIS3 wpl1D::LEU eco1D::KANMX6
